# An Electrochemistry and Computational Study at an Electrified Liquid–Liquid Interface for Studying Beta-Amyloid Aggregation

**DOI:** 10.3390/membranes13060584

**Published:** 2023-06-05

**Authors:** Bongiwe Silwane, Mark Wilson, Ritu Kataky

**Affiliations:** Department of Chemistry, Durham University, Durham DH1 3LE, UK

**Keywords:** beta-amyloid, electrified liquid–liquid interface, molecular dynamic simulations, aggregation, drug–peptide interactions, copper binding

## Abstract

Amphiphilic peptides, such as Aß amyloids, can adsorb at an interface between two immiscible electrolyte solutions (ITIES). Based on previous work (vide infra), a hydrophilic/hydrophobic interface is used as a simple biomimetic system for studying drug interactions. The ITIES provides a 2D interface to study ion-transfer processes associated with aggregation, as a function of Galvani potential difference. Here, the aggregation/complexation behaviour of Aβ_(1-42)_ is studied in the presence of Cu (II) ions, together with the effect of a multifunctional peptidomimetic inhibitor (P6). Cyclic and differential pulse voltammetry proved to be particularly sensitive to the detection of the complexation and aggregation of Aβ_(1-42)_, enabling estimations of changes in lipophilicity upon binding to Cu (II) and P6. At a 1:1 ratio of Cu (II):Aβ_(1-42)_, fresh samples showed a single DPV (Differential Pulse Voltammetry) peak half wave transfer potential (E1/2) at 0.40 V. Upon increasing the ratio of Cu (II) two-fold, fluctuations were observed in the DPVs, indicating aggregation. The approximate stoichiometry and binding properties of Aβ_(1-42)_ during complexation with Cu (II) were determined by performing a differential pulse voltammetry (DPV) standard addition method, which showed two binding regimes. A pKa of 8.1 was estimated, with a Cu:Aβ_1-42_ ratio~1:1.7. Studies using molecular dynamics simulations of peptides at the ITIES show that Aβ_(1-42)_ strands interact through the formation of β-sheet stabilised structures. In the absence of copper, binding/unbinding is dynamic, and interactions are relatively weak, leading to the observation of parallel and anti-parallel arrangements of β-sheet stabilised aggregates. In the presence of copper ions, strong binding occurs between a copper ion and histidine residues on two peptides. This provides a convenient geometry for inducing favourable interactions between folded β-sheet structures. Circular Dichroism spectroscopy (CD spectroscopy) was used to support the aggregation behaviour of the Aβ_(1-42)_ peptides following the addition of Cu (II) and P6 to the aqueous phase.

## 1. Introduction

Several neurodegenerative diseases are caused by amyloidogenesis, which occurs through the accumulation of amyloid-beta (Aβ) peptide as plaques and aggregates in the human brain. Aβ protein fibrils consist of 35–43 amino acid residues [1]. Both polymorphic oligomers and fully formed fibrillar aggregates of Aβ peptides are neurotoxic. It is currently believed that the smaller, soluble, oligomeric aggregates are more toxic than the full amyloid fibrils [2,3,4]. There are two hypotheses that describe the formation of amyloids plaques (the amyloid cascade hypothesis) [5] and soluble oligomeric aggregates (the oligomer hypothesis) [2]. Transition metal ions, such as copper, zinc and iron, are believed to be involved in the formation of aggregates, the misfolding of Aβ peptides and the formation of reactive oxygen species (ROS) [6]. Despite extensive studies, the mechanisms of metal and Aβ peptide are not fully understood [6,7,8,9,10,11].

**Scheme 1:**
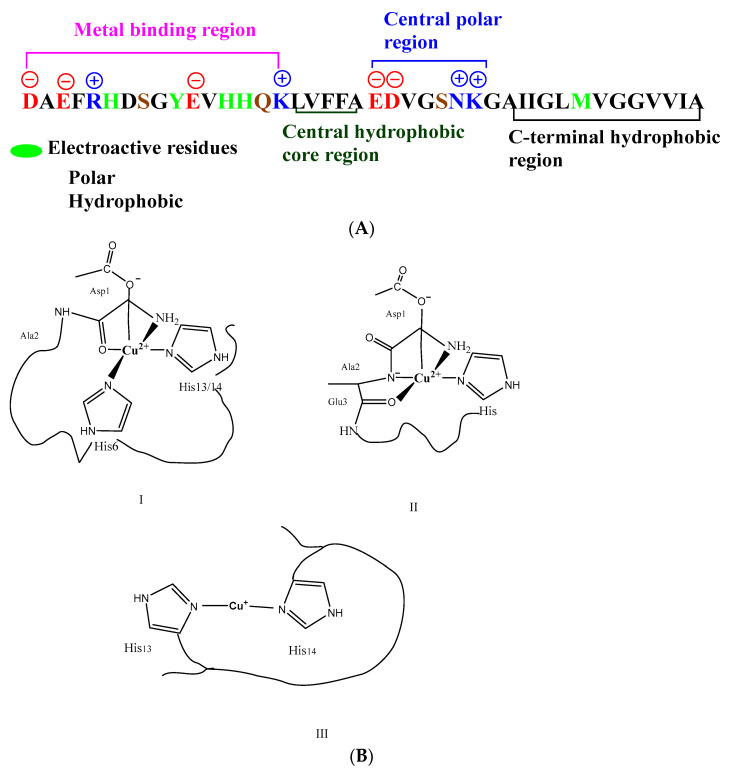

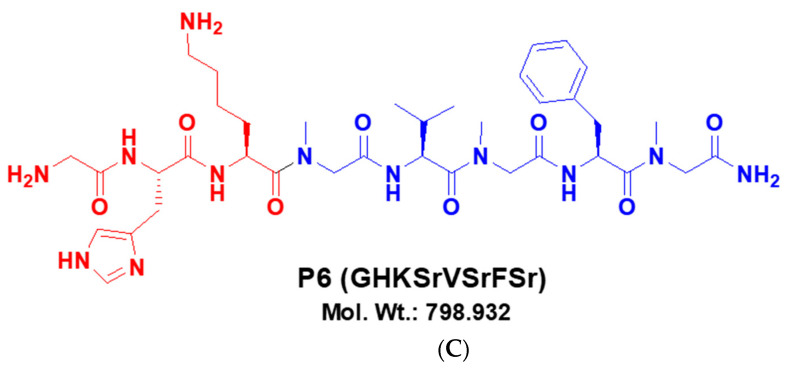
(**A**) Structure of amyloid-beta 1-42 and sequence showing electroactive amino acids and metal binding sites. (**B**) Possible Cu-Aβ complexes that are reported in literature. (**C**) Structure of peptidomimetic peptides (P6), P6 binds to Cu^2+^ through N-terminal glycine-histidine-lysine (GHK) groups and prevents its redox cycling in reducing conditions.

This work is focused on the long Aβ_1-42_ peptide sequence, which is reported to be present in the ratio 1:2 Aβ_(1-40)_:Aβ_(1-42)_ in amyloid plaques, and displays more toxic effects than the shorter sequences. Aβ_(1-42)_ (molecular mass 4514.08 Daltons) is amphiphilic (Scheme 1). It has six negatively charged residues (D1, E3, D7, E11, E22, D23) and three positively charged residues (R5, K16, K28), giving a net charge of −3 (sequence in Scheme 1A). Its isoelectric point is about 5.5. The molecule has a zeta potential of −35.6 ± 0.4 mV at a pH of 7.4 at 25 °C, with oligomers and fibrils as the major aggregates. The potential for Aβ self-aggregation arises from the Aβ sequence itself, which contains a hydrophobic C-terminal region and a largely hydrophilic N-terminal region [5]. As Aβ amyloids are amphiphilic, they distribute biphasically at liquid–liquid interphases. It has been suggested that this property of Aβ may be an additional factor for cytotoxicity by partial insertion into the plasma membrane initiating apoptosis [12,13].

Metal beta-amyloid interactions have been reported using a wide range of techniques ranging from histochemical staining to scanning electron microscopy with energy disperse X-ray analysis, secondary ion mass spectrometry (SIMS), laser ablation inductively coupled plasma mass spectrometry (LA-ICPMS) and NMR spectroscopy, amongst others [11,12]. In particular, Cu, Zn and Fe induced aggregation has been reported extensively, particularly with the shorter, soluble peptide fragment Aβ_1-16._ The conclusions of these reports are dependent on experimental conditions, and diverse results have been seen. Detailed discussions are beyond the scope of this paper.

The focus of the current work is on the role played by copper binding to Aβ_1-42,_ at physiological pH using a liquid–liquid interface. A brief account of the current views on this topic is included here [9,10,14]. Cu (II) can bind to beta-amyloids in dynamic coordination modes [15]. A likely structure for Cu^2+^ complexes with Aβ_1-16_ at a neutral pH, as reported in the literature, is shown in Scheme 1B with the involvement of three N donor atoms, (His6, His13/His 14 imidazole rings) and at least one O donor atom from a carboxyl or hydroxyl side chain, water/hydroxo or phosphate from buffers. The carboxylate ion in the apical position can interact with water via hydrogen bonding [16,17,18,19,20,21]. A reduced Cu^+^ complex has been proposed as a linear His13-Cu(I)-His14 motif [14]. However, with the longer sequences of Aβ peptides, there are reports of additional binding sites. Viles and co-workers [8] reported that two copper-binding sites are present in Aβ_(1-42)_ in a water/methanol mixture (80:20, *v*/*v*). Bush and co-workers [7] also showed two cooperative copper-binding sites in Aβ_(1-40)_ at a pH of 7.4. The coordination of the geometry of Cu to beta-amyloids is highly dependent on experimental conditions, such as pH, concentrations and temperatures, with a wide variety of reports on the exact coordinating amino acids. Diverse conclusions have been reached by different groups on the role of Cu (II) in the aggregation of beta-amyloids. In some reports, Cu (II) is reported to disrupt and reduce larger Aβ_1-42_ aggregates [16,17,18,19], whereas others report an enhancement of aggregation [20,21,22]. Additionally, Aβ_(1-40)_ was shown to form granular amorphous aggregates instead of amyloid fibrils at greater than equimolar Cu (II) ion concentrations [10]. The reader is referred to a review on the topic [12] for a more detailed discussion.

Several strategies have been proposed to intercept the formation of toxic beta-amyloid fibrils in the presence of redox-active metal ions. Recently, the work conducted by Rajasekhar and co-workers reported a multifunctional peptidomimetic inhibitor, P_6_, (Gly-His-Lys-Sr-Val-Sr-Phe-Sr) [23], with a pKa > than 9 which forms a 1:1 complex with Cu^2+^ (Scheme 1C). The molecule has a dual function of preventing the formation of Aβ oligomers and fibrils and sequestering Cu^2+^ from Aβ-Cu^2+^ complexes. P_6_, acts as an inhibitor of multifaceted Aβ toxicity by silencing the Aβ-Cu^2+^ redox cycle, which can generate Reactive Oxygen Species (ROS) through Fenton-type reactions. Further, it was shown, by employing various biophysical studies, that P_6_ interacts with Aβ and prevents the formation of toxic Aβ forms, such as oligomeric species and fibrillar aggregates. 

The amphipathic character of Aβ oligomers and fibrils suggests that the peptide will preferentially accumulate at the interface between two liquids with disparate dielectric constants. In this work, we exploit the fact that Aβ amyloids are amphiphilic and distribute biphasically at liquid–liquid interfaces, to study the interactions of Aβ_(1-42)_ with Cu (II) and P_6_ at an interface between two immiscible electrolyte solutions (ITIES). The ITIES provides a renewable interface for the study of ion transfer, adsorption and ion–molecule interactions that occur across the liquid–liquid interface induced by the interactions and the application of a potential difference between the two phases. Any charged chemical species is susceptible to transfer across the interface if the energy provided is sufficient [24]. Previously, ITIES was shown to facilitate the ion transfer of proteins and peptides with inherent hydrophobicities, even in the absence of organic phase ionophores to facilitate the transfer [25]. ITIES has a distinct advantage over traditional solid/liquid interfaces, as the liquid–liquid interface can be considered defect-free, which means that various reactants can be readily separated from one another and the direct interaction with a solid electrode phase is avoided. Amemiya and co-workers studied the behaviour of protamine, a cationic polypeptide with a charge of ca. +20, using μITIES [26]. Trojánek and co-workers [27] carried out studies of protamine at an aqueous-1,2-dichloroethane (DCE) macro interface and observed that the highly charged protamine interacted with the organic phase anions, as indicated by shifts in the forward and reverse peaks, in the cyclic voltammograms. Vagin and co-workers [28] studied the spontaneous formation of micelles at a liquid–liquid interface to study the detection of redox-inactive proteins. The surfactants adsorb specifically at the liquid–liquid interface and the ionic surfactants form micelles spontaneously at higher concentrations and transfer across the interface. The positive current observed with the anionic surfactant was attributed to the cation transfer by the formation of reverse micelles. Kievlehan, Lanyon and Arrigan [29] reported the behaviour of insulin at an aqueous/1,2 DCE interface using CV (Cyclic Voltammetry). The transfer at a pH at which insulin is cationic was dependent on the organic phase anion. Similar observations were made with studies using haemoglobin [25] and egg white lysozyme [30]. The transfer potential of each protein was dependent on the pH at which the protein was in the cationic form, allowing the protein to complex with hydrophobic anions and adsorb at the interface.

Importantly, ITIES can also be considered a model for understanding the interactions of amyloids with biological membranes. In cell membranes, the long hydrophobic tail (G29–V40 of A42) enhances membrane adhesion. This region is flanked by positively and negatively charged residues, KLVFFAE (=Aβ_(16-23)_) and K_28_ [12]. The metal binding His residues (H6, H13, H14), which are excellent ligands for copper, are located in the hydrophilic domain and are more accessible from the aqueous phase. The overall charge of the Aβ_1-42_-Cu complex significantly affects the surface charge at the liquid–liquid interface. At a physiological pH, Aβ_(1-42)_ has a charge of −3 due to 6 aspartic and glutamic acid residues and 3 lysine and arginine residues, assuming uncharged termini. The amyloid has a zeta potential of −35.6 ± 0.4 mV at a pH of 7.4 at 25 °C, which upon complexation with Cu^2+^, changes to −26.5 ± 0.3 mV, resulting in a change of lipophilicity.

In this work, the lipophilicity, binding and aggregation properties of Cu^2+^ with Aβ_(1-42)_ and the interaction with potential drug molecules, such as P_6_, are reported at a pH of 7.4. The conclusions from electrochemical experiments are supported by Circular Dichroism (Supplementary Materials, ESI S1). Atomistic molecular dynamics simulations are also performed to support our results on the assembly and aggregation properties of Aβ_(1-42)_ and its interactions with copper ions [9].

## 2. Experimental Section

### 2.1. Reagents and Chemicals

All the chemicals used were of analytical grade or better. Aβ_(1-42)_ human peptide was purchased from Discovery Peptide (Billingham, Cleveland, UK) and the stock solution (0.2 mM) was prepared with PBS (pH 7.4). The peptide solutions were sonicated and centrifuged to remove any aggregates. The solutions of the copper ions tested were prepared from the corresponding nitrate salts from Sigma (Gillingham, UK). LiCl (99.99%, Aldrich, Gillingham, UK) was used as the aqueous base electrolyte at a concentration of 10 mM, prepared in ultrapure water (resistivity of 18 MΩ cm) from a Milli-Q water purification system. The organic electrolyte salt was prepared by the metathesis of bis-(triphenylphosphoranylidene) ammonium chloride (BTPPA^+^Cl^−^) and potassium tetrakis (4-chlorophenyl) borate (K^+^ TPBCl^−^) to obtain BTPPATPBCl, following the well-known experimental procedure [28]. The organic electrolyte solution was prepared in 1,2-dichloroethane (1, 2-DCE, 5 mL, 99%, Sigma-Aldrich (Gillingham, UK). The organic reference solution consisted of 10 mM BTPPACl dissolved in 10 mM LiCl (aqueous). P_6_ ligand (multifunctional peptidomimetic inhibitor) was supplied by the Biochemistry Laboratory, Bangalore University (India).

### 2.2. Electrochemical

All voltammetry experiments were performed in triplicate, at room temperature, using a computer-controlled Autolab Potentiostat (Metrohm Autolab B.V., Utrecht, The Netherlands) with *iR* compensation together with the Nova 2.1 software supplied with the instrument. A four-electrode electrochemical cell, made in-house, was customised for the liquid–liquid voltammetry experiments. The interfacial potential difference was controlled using two Ag/AgCl electrodes (one in each phase). The current was measured using two Pt flag counter electrodes (one in each phase).

**Scheme 2:**
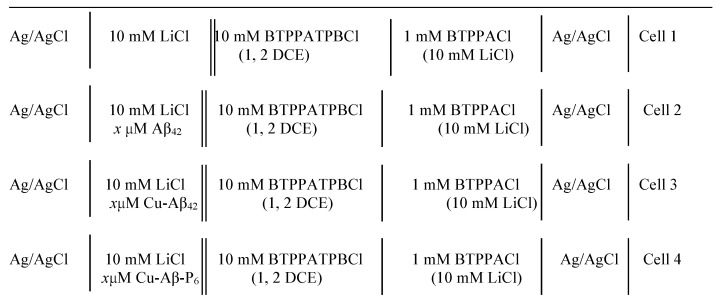
Configuration of the cells for the ITIES studies. x is the concentration ion in the aqueous phase. The double bar shows the polarised interface.

To investigate the interaction of Cu^2+^ with Aβ_(1-42)_ and Cu- Aβ_(1-42)_ with P_6_, the CV scans were recorded between 0 and 0.7 V vs. Ag/AgCl. For quantitative studies, Differential Pulse Voltammetry titration measurements were carried out by keeping the concentration and the volume of the Aβ_(1-42)_ in solution constant and varying the Cu^2+^ concentration. The DPV response of all the samples was recorded by scanning the potential from 0–0.7 V vs. Ag/AgCl. The experiments were carried out according to the following optimised DPV parameters: modulation time (s): 0.05; step potential (V): 0.005; modulation amplitude (V): 0.025; and interval time (s): 0.5. All the half-wave potentials for ion transfer were calculated by reference to the half-wave potential of the tetramethylammonium ion (TMA+). The electrochemical cells employed in this study are summarised in Scheme 2. In the CVs, positive currents are attributed either to the transfer of anions from the organic phase to water, or to the transfer of cations in the reversed direction and vice versa for negative currents.

Circular Dichroism (CD) spectroscopy was carried out with samples removed from the aqueous phase of the 1,2 DCE/ Aqueous interface containing the respective electrolytes, prepared so as to replicate the cell in Scheme 2. Experimental details and analysis are available in Supplementary Materials ESI: (S1).

### 2.3. Computational

Molecular dynamics simulations were undertaken using the GROMACS 2018.7 suite of programs [31], using the GROMOS 53a6 force field for proteins [32] together with SPC water and NaCl (see below for details). For simulations at a hydrophilic–hydrophobic interface, a united atom model for 1,2-dichloroethane (DCE) was used, based on the GROMOS 53a6 parameter set (see Supplementary Materials ESI S2). The simulations were, typically, pre-equilibrated over a few ns using the canonical (constant-*NVT*) ensemble, and immediately followed by a few ns of simulation within the isobaric–isothermal ensemble (constant-*NpT,* Berendsen thermostat and barostat) to remove any close contacts. The simulations were then fully equilibrated in the isobaric–isothermal ensemble at 300 K, employing a Nosé–Hoover thermostat [33,34] and a Parrinello-Rahman barostat [35,36] at atmospheric pressure and using isotropic pressure coupling. The bond constraints were applied using the LINCS algorithm [37] with a 2 fs time step. Interaction cut-offs were applied for Lennard-Jones (1.2 nm) and Coulombic interactions (1.2 nm). The long-range part of the Coulomb potential was accounted for by employing a Particle Mesh Ewald (PME) summation [38,39].

Peptide coordinates for the Aβ_1-42_ peptide were originally obtained from the RCSB Protein Data Bank (PDB structure reference 1IYT [40]) and solvated in simple point charge (SPC) water with ~10 mM NaCl. We follow Strodel et al. [41] and model the histidines as uncharged, resulting in a total charge for Aβ_1-42_ of −3, which was neutralised by additional sodium ions. The initial peptide secondary structure contained two α-helices. The initial equilibration of this structure over 40 ns in SPC water led to the loss of both α-helices and the formation of a beta-hairpin structure for residues 30–41 (see Supplementary Materials ESI S3), which was used in subsequent simulations.

To test the stability of the peptide at a hydrophobic–hydrophilic interface, a water box containing the peptide was combined with a DCE box (see ESI S6 for details) and a 10 ns simulation was carried out to show the capture of the peptide by the interface. The whole simulation box was then duplicated in *x* and *y* directions (giving four protein molecules at the interface), some solvent molecules were removed (to reduce the overall system size) and the system was compressed to control the interface cross-section (in the *x*, *y* plane), prior to further equilibration (see ESI S6 for details). Production runs were carried out for simulations of four peptide molecules in boxes with average dimensions of 6.25 nm × 6.16 nm × 61.56 nm (box cross-sections of 38.5 nm^2^) and 9.51 nm × 9.53 nm × 14.50 nm (box cross-section of 90.7 nm^2^), and (after deleting two proteins) for a protein dimer in a box with average dimensions of 9.95 nm × 9.94 nm × 13.19 nm (box cross-section of 98.9 nm^2^).

The previous literature studies [9,10,14,15,16,17,18,19,20,21] have looked at the influence of copper (II) ions on secondary structure. The copper binds strongly to three histidine residues and a carboxylic acid (e.g., from the Glu11 or Asp1 residue [42]). This provides a six-coordinate distorted octahedral structure (Supplementary Materials, ESI S5) with space for the binding of a water molecule at the sixth coordination site. When the binding of Cu^2+^ occurs to just one protein strand, previous simulations have suggested that the formation of β-sheet structures is disrupted [43]. Barnham and Bush [7] and Viles et al. [8] have suggested, however, that copper ions can link β-sheet structures from two chains, binding His (13) and His (14) on one chain and His (6) on a second chain. This has the potential to enhance the aggregation of β-sheet structures [1] and provides an explanation for increases in the unbinding force between two Aβ_1-42_ peptides in the presence of Cu^2+^, as measured by the single molecule AFM.

We also considered the possibility that copper can be present in reduced form (4-coordinate) Cu^+^ ions. Here, we calculated the structure of a likely copper (I) binding site using density functional theory at the B3LYP/LANL2DZ level. Calculations were undertaken for four-coordinate copper (I), with three histidines and a carboxylic acid group. This binding site is shown in the Supplementary Materials, ESI S5, where it is compared to the known distorted octahedral binding site of copper (II).

For copper systems, explicit bonding potentials were added to the proteins to provide permanent bonds between histidine and carboxylic acid groups and copper ions in the molecular dynamics simulations. In this work, we follow Hane et al. [1] and bond the copper to two protein chains: specifically, the His13 and His14 for chain1 and His6 for chain 2 (as discussed below). Additional force field parameters are given in the Supplementary Materials (ESI S6).

To introduce a copper ion to the protein simulations, a Na^+^ ion was converted to a copper ion (with an additional Na^+^ ion deleted for Cu^2+^). Initially, restraints were added to move the ion close to the coordinated nitrogens on His13 and His14 using a very weak force constant of 10 kJ mol^−1^ nm ^−2^. This was gradually increased to 3 × 10^5^ kJ mol^−1^ nm^−2^ over a series of short simulations of the length 20–100 ps, and then replaced by a LINCS bond constraint. The process was repeated to introduce a bond to a nitrogen on the His6 residue of a second chain. At the end of this process, the carboxylic acid group from Glu11 (chain 1), as suggested in the XAS work of Streltsov et al. [44], was in close proximity to the copper ion and was chosen for the final coordination site(s). The structure of the copper bonding sites was in good agreement with the DFT structures (see ESI S5). The final structures were equilibrated, and production runs were carried out in a box of cross-section ~9.8 nm × 9.8 nm. A summary table with the systems simulated is included (ESI S6).

## 3. Results and Discussion

In a previous study, Nichols and co-workers [13] showed that the Aβ_(1-42)_ peptide accumulates preferentially at a hydrophilic/hydrophobic interface when it is introduced to an aqueous buffer in a two-phase system with chloroform. The peptide was shown to aggregate much more rapidly at a liquid–liquid interface than in the buffer alone. The kinetics of aggregation exhibits, on a macroscopic level, three characteristic stages: a lag phase, a growth phase and a final plateau regime. The characteristic lag phase, prior to onset of aggregation, is reduced from several weeks to hours at a liquid–liquid interface. The interface-induced aggregates were released into the aqueous phase and persisted for 24–72 h before settling as a visible precipitate at the interface.

The Circular Dichroism spectroscopy was performed using samples removed from the aqueous phase of 1,2 DCE/Aqueous interface containing the respective electrolytes, prepared to replicate the cell in Scheme 2 (ESI S1). The spectrums, after 24 h incubation, confirmed the inhibition of Aβ_42_-Cu (II) aggregation upon the addition of P6 (for details, see ESI S1 in Supplementary Materials).

### 3.1. Computational 

**Figure 1:**
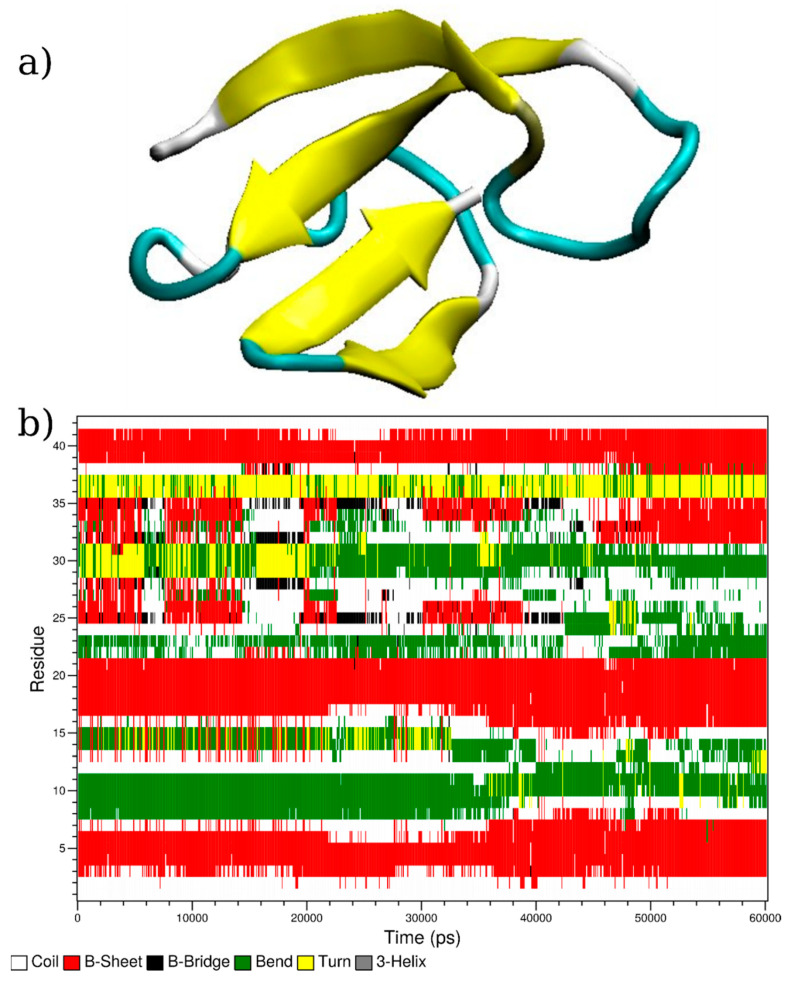
(**a**) Snapshot of the folded structure of Aβ_1-42_ taken from the end of a 285 ns production run in water. (**b**) Protein secondary structure during the final 60 ns of the simulation, determined using the DSSP software [46,47].

Molecular dynamics simulations (285 ns) of Aβ_1-42_ in water show the spontaneous formation of a β-sheet folded structure (Figure 1), which is believed to aid aggregate formation. As discussed above, the presence of a hydrophobic–hydrophilic interface has been shown to enhance the rate of aggregation. We hypothesise that: (i) Aggregation is aided through the initial confinement of peptide chains to the interface, which increases the interaction between individual peptide chains. (ii) The latter is aided by β-sheet formation, which provides a mechanism for increasing *n*-mer aggregation. (iii) Binding copper (II) between peptide dimers enhances this process. We tested these hypotheses with further simulations.

We note in passing that recent work [45] has indicated that even short peptides can require run lengths well in excess of 300 ns to fold. However, in the case of Aβ_1-42_, the structure transforms quickly and completely from the initial PDB secondary structure containing two α-helices (see Figure S3a, which was obtained as a 3D NMR structure in an apolar solution) to a β-sheet folded structure in SPC water. Hence, it is highly likely, in this case, that the simulations faithfully capture the driving force for β-sheet formation within SPC water.

**Figure 2:**
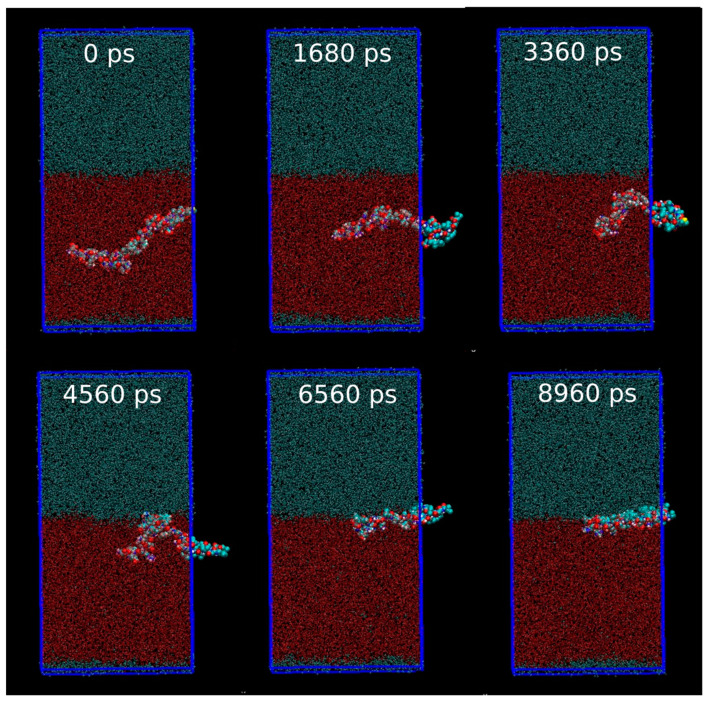
Snapshots from a 10 ns run showing the capture of the Aβ_1-42_ peptide by a hydrophobic–hydrophilic interface, water molecules are shown in red (dots) and DCE molecules are shown in blue/gray (dots).

Figure 2 shows the capture of the peptide by the hydrophobic–hydrophilic interface. Initially, the peptide was free to diffuse in the water layer, until part of the peptide came into contact with the interface at approximately 4.5 ns into the simulation. The rest of the peptide was then quickly captured by the interface over a further period of ~2 ns. Thereafter, the peptide remained at the interface for the remainder of the 10 ns simulation, and also for subsequent simulations, while exhibiting free diffusion in the *x*, *y* plane.

**Figure 3:**
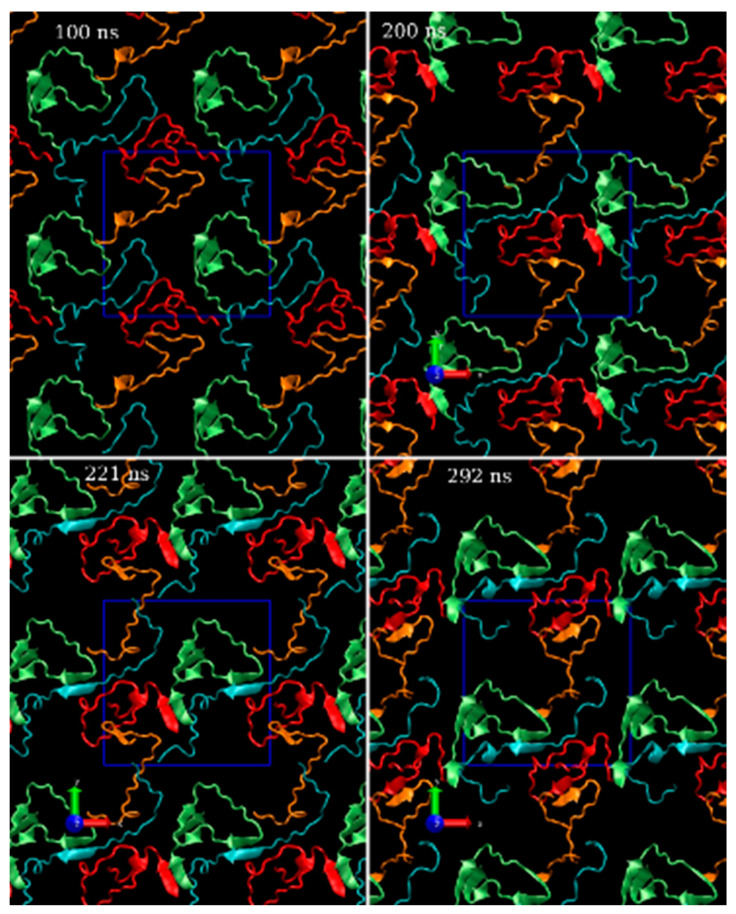
Snapshots showing aggregation of Aβ_1-42_ peptides at a DCE–water interface. Four chains are shown in cyan, red, lime green and orange together with protein secondary structure. The dark blue box outline indicates the 2D profile of the periodic box, which shows an average dimension of ~6.25 nm × 6.15 nm.

**Figure 4:**
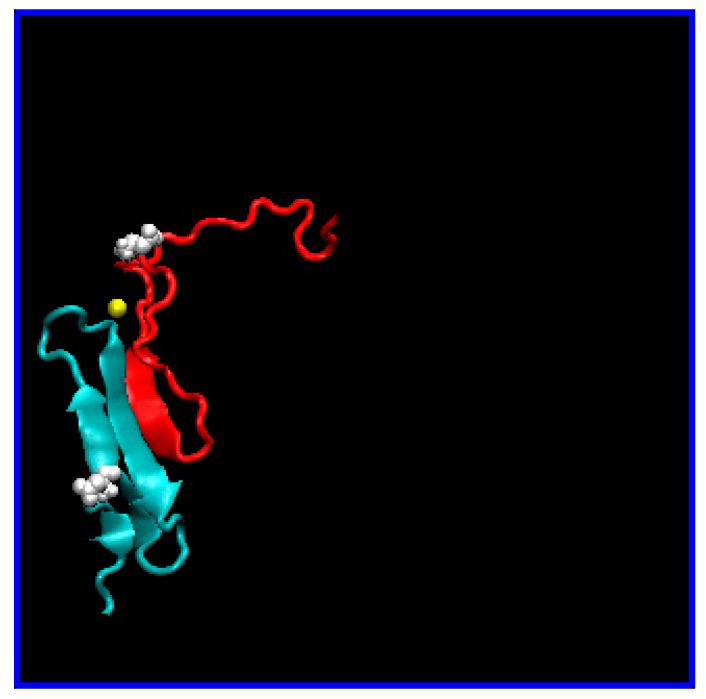
Association of an Aβ_1-42_ peptide dimer bound by copper at the dicholoroethane-water. The picture shows a snapshot form the end of a 1470.9 ns molecular dynamics run with a copper (II) ion linking two chains (as shown in Figures S3b and S4 (see ESI)). Individual chains are colour-coded in cyan and red, the copper ion is shown in yellow and the first residue of each chain is shown in white. The area of the interface is 9.95 nm × 9.94 nm.

The aggregation of peptides at the liquid–liquid interface occurs via the formation of β-sheet structures, and then, subsequently, the strong interactions between these. Figure 3 and Figure S9 (see ESI) show peptide aggregation for two separate simulation runs at different surface concentrations. In the absence of copper, both anti-parallel and parallel interactions occur between β-sheet structures, and these are seen at different points during the simulation. The propensity to aggregate is quite strong even at a low surface concentration, where the proteins form a disordered network (Figure S9). In Figure S8, we compare the behaviour of a protein dimer without copper and with bound copper (I), or copper (II) ions linking two peptide chains. A comparison is made over long simulation runs (1.20 μs, 1.66 μs, 1.47 μs, respectively) where we see both parallel and anti-parallel arrangements of β-sheets that change dynamically over the course of the simulation. However, the binding of copper (II) ions is seen to strongly promote the growth of anti-parallel packed β-sheet structures (see Figure 4). As suggested by the AFM work of Hane et al. [1], this is likely to provide a mechanism for the rapid growth of aggregates through the strong interactions arising from the inter-chain β-sheet stacking of peptide dimers.

### 3.2. Electrochemical Measurements

#### 3.2.1. Cyclic Voltammetry at the Liquid–Liquid Interface

**Figure 5:**
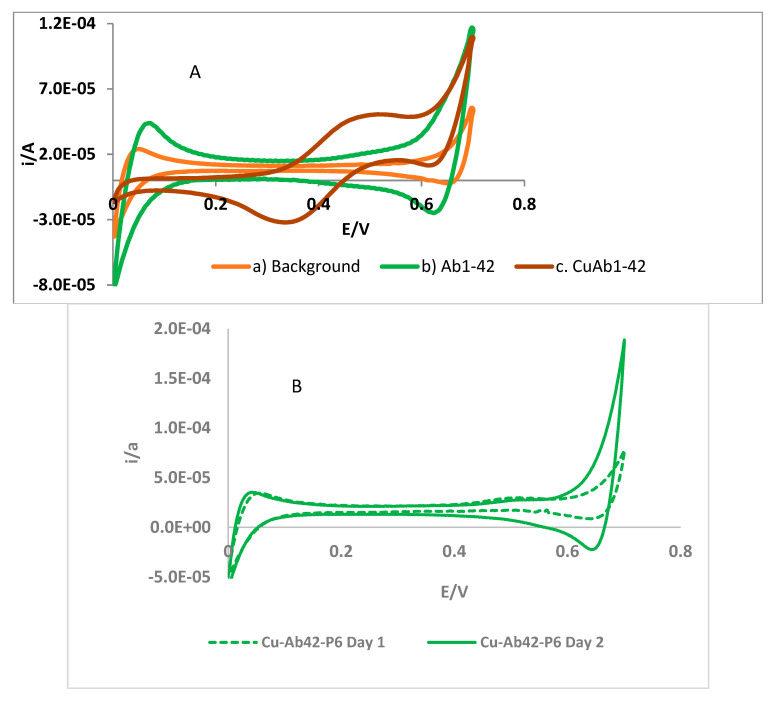
(**A**): CVs of (a) background solutions (b) with 0.01 M Aβ_(1-42)_ added to the aqueous phase (c) with Cu(II)-Aβ_(1-42)_, 1:1 ratio in the aqueous phase, corresponding to the cells shown in Scheme 2, Cell 1, 2 and 3, using a scan rate 0.1 V/s. (**B**): CV of Cu-Aβ and P6, 1:4 ratio, using a scan rate 0.1 V/s at ITIES after 10 m and after 24 h.

An electrified liquid–liquid interface was used to study the complexation and aggregation behaviour of Aβ_(1-42)_, Cu^2+^ and P_6_. The amphiphilic Aβ_(1-42),_ which consists of a hydrophilic N-terminal domain (1–28), containing the metal binding site, and a C-terminal hydrophobic domain (29–40/42), is expected to migrate towards the interface. Both the aqueous and non-aqueous phases contain added electrolytes; it is, therefore, important to study the effect of the electrolyte ions on the peptide. Observations from CVs performed using Cell 1 (Scheme 2) indicate that within the potential range 0.13–0.65 V, the liquid–liquid interface boundary behaves as an ideally polarised interface with the background electrolyte solutions (Supplementary Figure S6). Upon the addition of 0.01 μM Aβ_(1-42)_ to the aqueous phase (Cell 2, Scheme 2), the CV retains a similar shape (Figure 5Ab) within the time frame of the experiment. Increasing the scan rate (Supplementary Figure S4) results in both the positive peak and negative peak heights increasing. The peak potential for the negative and positive peak shifts from 0.65 V and 0.07 V (at 15 mV/s scan rate) to 0.61 V and 0.13 V (at 100 mV/s scan rate). In previous studies, cationic polypeptides were shown to complex with the organic phase anion and the anion capture and release at the interface resulted in a shift in the positive and negative peaks. Unlike these cationic peptides, Aβ_(1-42)_ is anionic with a charge of −3, so it is likely to interact with the organic phase cations. However, the cations in the electrochemical cells, which are the highly lipophilic BTTPA^+^ in the organic phase and Li^+^ in the aqueous phase, are not known to complex with beta-amyloids. We believe that this results in a relatively flat voltage window between 0.13 V and 0.57 V.

CV measurements were performed for monitoring the complexation of Cu^2+^ to Aβ_(1-42)_ at a 1:1 ratio (Figure 5Ac). A positive peak is observed at 0.50 V (vs. Ag/AgCl) in the forward scan and two peaks at 0.63 V and 0.35 V (vs. Ag/AgCl) at the reverse negative scan. Scan rate studies (ESI, Figure S5) indicate that the positive peak current at 0.50 V and the negative peak at 0.35 V vary linearly with the square root of the scan rate, typical of a diffusion-controlled process. A negative peak observed at 0.63 V varies with the scan rate. This peak could be attributable to a species that is a metastable intermediate shown in a ‘pre-organisation’ mechanism identified by Saveant and co-workers, which relaxes to the stable form of the Cu^+^ complex [48]. Upon the addition of an excess of the P6 at a 1:4 proportion (Figure 5B), a decrease in the CV peaks is evident with a further decrease on Day 2, indicating the breakdown of the Aβ_(1-42)_ Cu aggregates.

These initial qualitative results confirm the strong interaction of Cu ions with Aβ_(1-42)_ and the effectiveness of P6 as a multifunctional peptide inhibitor.

#### 3.2.2. Differential Pulse Voltammetry (DPV) at the Liquid–Liquid Interface

Differential pulse voltammetry was used to evaluate the aggregation behaviour and complexation of Aβ_(1-42)_, P_6_ and Cu^2+^. DPV allows Faradaic currents to be accessed more readily by decreasing the contribution of charging currents. The kinetics and thermodynamics of the redox reactions of Cu-Aβ_(1-42)_ at a liquid–liquid interface are complex and challenging. The transition from the ordered fibril formation of the beta-amyloid to the formation of amorphous aggregates, particularly at higher concentrations of metal ions, further complicates the analyses.

**Figure 6:**
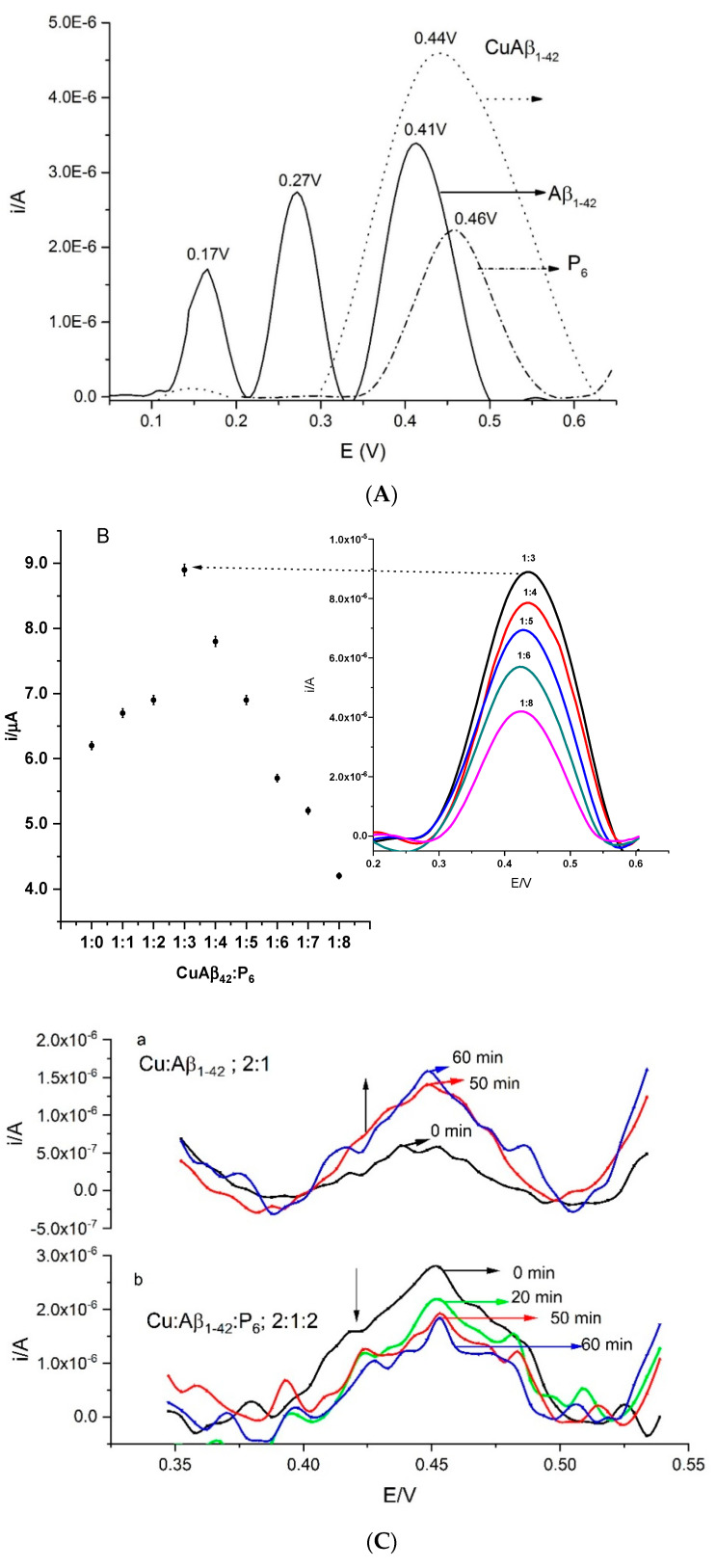
Background, phosphate buffer, subtracted DPVs. (**A**): Solid line (Cell 2, Scheme 2): Aβ_1-42_. Dotted line, CuAβ_1-42_ 1:1 (Cell 3, Scheme 2). Dashed line, CuAb_1-42_; P6 1:4 (Cell 4, Scheme 2). (**B**): Effect of addition of 0.5 µM P6 to the 0.5 µM CuAβ_1-42_ complex formed freshly. The signal starts decreasing when the ratio of P6 reaches 1:3. (**C**): Effect of excess Cu^2+^; (**a**): 0.02 μM Cu:0.01 μM Aβ_(1-42)_ in a ratio 2:1 showing increasing current with time. The fluctuations in current indicate aggregation phenomenon at the liquid–liquid interface. (**b**): DPV obtained upon addition of 0.03 μM P6, showing a decrease in current with time, (**B**).

At a physiological pH, DPVs at the ITIES showed three distinct peaks at potentials 0.17 V, 0.27 V and 0.41 V vs. Ag/AgCl, (Figure 6A, solid line) for the peptide Aβ_(1-42)._ This is likely because of the effects of the liquid–liquid interface on the structure and conformation of the peptide. Zhai and co-workers [49] reported the significant unfolding of the aromatic residues of the hydrophobic core of the globular protein α-lactalbumin upon adsorption at oil–water interfaces. In contrast, the peptide (P_6_) shows a single peak at 0.46 V vs. Ag/AgCl (Figure 6 dashed line). Upon the addition of Cu (in a 1:1 ratio) to the aqueous phase, a single significant peak at 0.44 V was observed (Figure 6 dotted line). We believe this peak is due to the transfer by the interfacial complexation of Aβ_1-42_ with the copper ion:

$$n{\left(A\beta_{1-42}\right)}^{3-}(interfaca)+Cu^{2+}\begin{array}{c} K^{o}\\ ⇌\\ K^{aq} \end{array}{\left(A\beta_{1-42}Cu\right)}^{3n-2}$$

where the transfer species is dictated by the Gibbs free energy of transfer. The association constants. The number of ligands ‘n’ can be 1 or 2.

The effect of the P6 on the CuAβ_1-42_ complex was then monitored using increasing concentrations of the molecule (Figure 6B). The current intensity was observed to decrease when the ratio of the Cu-Aβ_(1-42)_ complex to P6 was 1:3, indicating the sequestering of Cu^2+^ by P6 and breakdown of Cu-Aβ_(1-42)_ aggregates. This decrease continues even when the ratio reached 1:8. Govindaraju and co-workers^26^ previously reported that a 1:5 (Aβ_(1-42)_:P6) ratio exhibited improved inhibition efficiency, but beyond this concentration, the inhibition efficiency did not improve.

Significantly, there was a difference in the DPVs obtained upon the addition of Cu^2+^ to the aqueous phase in a 2:1 ratio, and the DPV showed several smaller peaks which are probably due to the instantaneous formation of amorphous aggregates, which are known to form with higher concentrations of Cu. The work conducted by Jiang et al. [50] has shown that the Cu:Aβ ratio is a major determinant of the aggregation pathway. They identified three different kinetic pathways that Aβ_(1-42)_ may take under the influence of Cu^2+^. The first pathway, where [Cu] < [Aβ], results in both fibrillary and oligomeric formation with higher copper concentrations resulting in higher proportions of oligomeric forms of amyloid-β [51]. An overall increase in the peak current was observed. Upon the addition of the copper chelator, P_6_, the current decreased with time (Figure 5B and Figure 6A) due to the sequestering of Cu (II).

**Figure 7:**
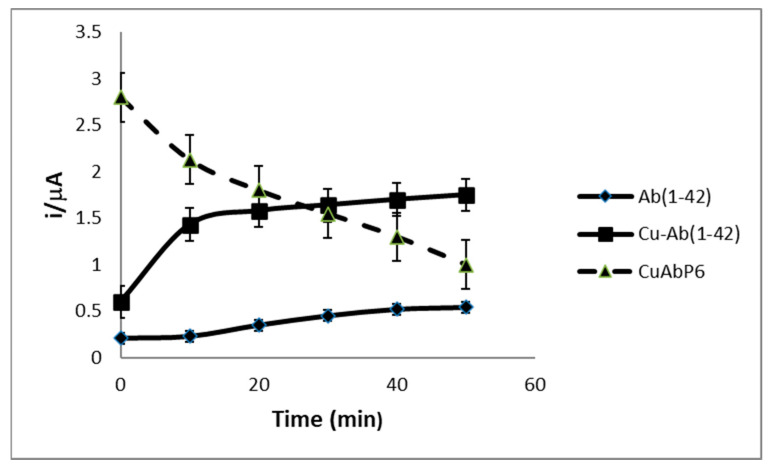
Summary of the effect of time (0–60 min) on changes in current, at a potential of 0.46 V, observed at the electrified liquid–liquid interface for Aβ_(1-42)_, 1:1 Aβ_(1-42)_ Cu complex and upon addition of P_6_ to the complex at an Aβ_(1-42)_ Cu:P6: 1:3 ratio.

Figure 7 summarises the effects of incubation time on Aβ_(1-42)_ and the comparative differences in the behaviour of the 1:1 Cu:Aβ_1-42_ complex upon the addition of P_6_ when the potential is held at 0.46 V at the ITIES. The time dependence of Aβ_(1-42)_ showed an approximately constant current from 0 to 10 min with a steady increase up to 40 min, followed by a plateau. These three regions may be attributable to the three characteristic stages of aggregation: a lag phase, a growth phase and a final plateau regime. With a Cu:Aβ_1-42_: 1:1 ratio, a sharp increase in the current intensity up to 10 min was observed, followed by a gradual increase for about 60 min. Upon the addition of P6 to Cu-Aβ_(1-42)_, in a ratio 1:3, Cu:Aβ_1-42_:P6, an increase in the current was initially observed, followed by a decrease with time. Taking into account previous reports and our work described above, showing that at least a threefold excess of the copper binding ligand P6 was required for the effective sequestering of Cu (II) (Figure 6B), this observation suggests a time-dependent sequestering of Cu (II) and breakdown of aggregation at a lower drug ratio of 1:1 Cu-Aβ_(1-42)_ complex:P6.

### 3.3. Binding Constant and Lipophilicity Estimates

The changes in the lipophilicities of the Cu-Aβ_(1-42)_ complex and upon the addition of P_6_ were obtained by calculating the partition coefficient of the complex across the interface using Equation (1) (Table 1):

(1) $$\log\;P_{i,w}^{0}=-\frac{Z_{I}\;F\Delta_{w}^{0}\emptyset_{i}}{RT\ln10}$$

where log $P_{i,w}^{0}$ is the standard partition coefficient of species *i*, $\Delta_{w}^{o}\phi_{i}$ is the formal transfer potentials of the species, from the aqueous to the organic phase, *R*, *F* and *T* are the gas constant, Faraday’s constant and temperature in Kelvin, respectively. The standard potentials were determined from the half-peak potential and the half-peak potential of the internal standard, tetramethylammonium ($\Delta \emptyset_{TMeN^{+}}^{DCE}=-0.160\;V)$, using the equation:

(2) $$\Delta_{w}^{o}\phi_{i}=\Delta_{w}^{o\;}\phi_{i}^{1/2}\;–\;\Delta_{w}^{o}\Delta_{i\;ref}^{1/2}+\Delta_{w}^{o}\phi_{ref}^{o}\;$$

The lipophilicity of the Cu Aβ_(1-42)_ aggregates increases more than tenfold in approximately 10 min from a Log$P_{w/o}^{0,i}$ of approximately 4.0 to 11.2, and then decreases to 3.1 upon the addition of excess P6, showing a significant decrease.

In order to investigate the complexation of Cu^2+^ with Aβ_(1-42)_ at the liquid–liquid interface, a titration was performed through the addition of a varying concentration of Cu^2+^ to a fixed concentration of Aβ_(1-42)_ in the aqueous phase. The concentration of the added Cu (II) was limited to a sub-stoichiometric ratio just below 1:2 of Aβ_(1-42)_:Cu (II) to avoid fluctuations due to aggregation. The analysis of the signals is complex and ambiguous due to the heterogeneity of the sites and aggregation of the complexes. Nevertheless, the data point up to a concentration ratio of Cu (II):Aβ_(1-42)_ < 2:1, indicating that the reactivity of Cu (II) with Aβ_(1-42)_ proceeds in two or possibly three stages, at a liquid–liquid interface. The Scatchard plots of Cu (II) interactions with Aβ_(1-42)_ were constructed using the experimental DPV curves. The Scatchard plot (Figure S7) clearly shows a nonlinear plot, indicating two binding sites with possibly positive and negative cooperativity, which would indicate binding followed by aggregation [52]. An approximate estimate of the association constant and binding stoichiometry for the most significant complex gives a value of pK_a_ 8.1 and a binding ratio *r* ~1.7 value (R^2^ = 0.99) (Fitting Shown in Figure S6).

These findings are in reasonable agreement with previous publications. The literature reveals a diversity of models dependent on pH and experimental conditions. Component 1a (Scheme 1B) is proposed at the predominant species at a lower pH [48], whereas the predominant coordination mode at pH 7.4 involves the residues Ala2, His6, His13 and His14 (Scheme 1B, component 2). Ghosh and co-workers [15] proposed an equilibrium between these two species in the range of pH 6.5–9.5, with a pKa of approximately 8.1. Streltsov and co-workers [44] reported that in phosphate-buffered saline, the salt concentration does not affect the high-affinity copper binding mode, but alters the second coordination sphere. A distorted six-coordinated (3N3O) geometry around copper in the Aβ-Cu^2+^ complexes was observed, which included three histidines, glutamic, or/and aspartic acid and axial water (ESI: Figure S4). This structure is consistent with the hypothesis that the redox activity of the metal ion bound to Aβ_(1-42)_ can lead to the formation of dityrosine-linked dimers found in Alzheimer’s diseases.

Theoretical studies showed that Aβ_(1-42)_ binds to several regions, including the N-terminal, central hydrophobic core and the C-terminal regions [53]. Atwood and co-workers [10] showed that Cu^2+^ binds at a 2:1 and 2.5:1 ratio with Aβ_(1-42)_, with a binding constant of 1.9 × 10^8^ and 3.7 × 10^7^, respectively. In further studies, Jun and co-workers used a combination of transmission electron microscopy (TEM), atomic force microscopy (AFM) and electron spin echo envelope modulation (ESEEM) spectroscopy to prove two binding regimes [54]. At equimolar concentrations of Cu (II) ions and fibrillary structures of Aβ_(1-40)_ in an N-ethyhlmorpholine buffer, Cu (II) was found to coordinate by histidine residues. However, aggregated Aβ_(1-40)_ at a Cu (II):Aβ molar ratio of 2:1, TEM and AFM images showed both linear fibrils and granular amorphous aggregates, and ESEEM spectra showed that the multi-histidine coordination for the Cu (II) ion partially breaks up and reveals a second Cu (II) binding site in the vicinity of water and exchangeable peptide protons. This resulted in the granular, amorphous aggregates, which they observed with AFM and which this work shows in the DPV studies.

## 4. Conclusions

This work illustrates that a liquid–liquid interface provides a simple model to reveal information on aggregation, binding and the effect of chelation and aggregation inhibitors on long-chain amyloid peptides. A combination of modelling and experimental techniques was used to study the behaviour of the most toxic form of beta-amyloid, Aβ_1-42_, at an electrified liquid–liquid interface, taking advantage of its amphiphilic properties. The binding of copper ions was investigated, followed by the effect of a multifunctional peptidomimetic inhibitor (P6). Molecular dynamics simulations show the capture and confinement of a single Aβ_1-42_ chain at the liquid–liquid interface and show that there is a strong propensity for chains to aggregate at the interface through the formation of parallel and anti-parallel stacked β-sheet structures. The binding of copper (II) ions between two chains is seen to promote anti-parallel β-sheet stacking, which provides a mechanism for increasing the interaction between dimers and hence promoting aggregate growth, as suggested in the earlier AFM work of Hane et al. [1].

Electrochemical methods at an ITIES support the formation of a major complex of Aβ_1-42_ with Cu (II), with a stoichiometry of Cu:Aβ_1-42_, at 1:1.7. The pKa of this complex is approximately 8.1. Additionally, studies of the lipophilicity changes of the Aβ_1-42_ at the ITIES, with the addition of Cu (II) ions and P6, show distinct trends. There is a significant increase in the lipophilicity upon binding with Cu (II) ions. The lipophilicity decreases upon the addition of P6 supporting the efficacy of the pepidomimetic P6, which binds with Cu (II) and suppresses aggregation.

Theoretical studies and electrochemical methods at a liquid–liquid interface in tandem provide an effective means of studying aggregation, lipophilicity behaviours and the effectiveness of peptides and peptide aggregation inhibitors.

## Figures and Tables

**Table 1:** Lipophilicities at the ITIES, calculated using Equations (1) and (2).

Complexes	$\Delta G_{tr,i}^{0,w}\left(kJ/mol\right)$	$\Delta_{O}^{W}\phi_{i\;\;}^{0}\left(V\right)$	$\mathrm{Log}P_{w/o}^{0,i}$
Cu-Aβ_42_	−21.11 ± 0.06	−0.22 ± 0.07	4.04 ± 0.3
Cu-Aβ_42_ (>10 min)	−58.86 ± 0.07	−0.61 ± 0.09	11.27 ± 0.5
Cu-Aβ-P_6_	−16.40 ± 0.07	−0.17 ± 0.09	3.14 ± 0.2

## Data Availability

Not applicable.

## Supplementary Materials

The following supporting information can be downloaded at: https://www.mdpi.com/article/10.3390/membranes13060584/s1.

## References

[1] Hane F., Tran G., Attwood S.J., Leonenko Z. (2013). Cu^2+^ Affects Amyloid-β (1-42) Aggregation by Increasing Peptide-Peptide Binding Forces. PLoS ONE.

[2] Lesné S., Koh M.T., Kotilinek L., Kayed R., Glabe C.G., Yang A., Gallagher M., Ashe K.H. (2006). A Specific Amyloid-β Protein Assembly in the Brain Impairs Memory. Nature.

[3] Sharma A.K., Pavlova S.T., Kim J., Finkelstein D., Hawco N.J., Rath N.P., Kim J., Mirica L.M. (2012). Bifunctional Compounds for Controlling Metal-Mediated Aggregation of the Aβ_42_ Peptide. J. Am. Chem. Soc..

[4] Sharma A.K., Pavlova S.T., Kim J., Kim J., Mirica L.M. (2013). The Effect of Cu^2+^ and Zn^2+^ on the Aβ_42_ Peptide Aggregation and Cellular Toxicity. Metallomics.

[5] Hardy J.A., Higgins G.A. (1992). Alzheimer’s Disease: The Amyloid Cascade Hypothesis. Science.

[6] Zhu X., Su B., Wang X., Smith M.A., Perry G. (2007). Causes of Oxidative Stress in Alzheimer Disease. Cell. Mol. Life Sci..

[7] Barnham K.J., Bush A.I. (2008). Metals in Alzheimer’s and Parkinson’s Diseases. Curr. Opin. Chem. Biol..

[8] Viles J.H. (2012). Metal Ions and Amyloid Fiber Formation in Neurodegenerative Diseases. Copper, Zinc and Iron in Alzheimer’s, Parkinson’s and Prion Diseases. Coord. Chem. Rev..

[9] Sarell C.J., Syme C.D., Rigby S.E.J., Viles J.H. (2009). Copper(II) Binding to Amyloid-β Fibrils of Alzheimer’s Disease Reveals a Picomolar Affinity: Stoichiometry and Coordination Geometry Are Independent of Aβ Oligomeric Form. Biochemistry.

[10] Atwood C.S., Scarpa R.C., Huang X., Moir R.D., Jones W.D., Fairlie D.P., Tanzi R.E., Bush A.I. (2000). Characterization of Copper Interactions with Alzheimer Amyloid β Peptides. J. Neurochem..

[11] Rana M., Sharma A.K. (2019). Cu and Zn Interactions with Aβ Peptides: Consequence of Coordination on Aggregation and Formation of Neurotoxic Soluble Aβ Oligomers. Metallomics.

[12] Rauk A. (2009). The Chemistry of Alzheimer’s Disease. Chem. Soc. Rev..

[13] Nichols M.R., Moss M.A., Reed D.K., Hoh J.H., Rosenberry T.L. (2005). Rapid Assembly of Amyloid-β Peptide at a Liquid/Liquid Interface Produces Unstable β-Sheet Fibers. Biochemistry.

[14] Hureau C., Balland V., Coppel Y., Solari P.L., Fonda E., Faller P. (2009). Importance of Dynamical Processes in the Coordination Chemistry and Redox Conversion of Copper Amyloid-β Complexes. J. Biol. Inorg. Chem..

[15] Ghosh C., Dey S.G. (2013). Ligand-Field and Ligand-Binding Analysis of the Active Site of Copper-Bound Aβ Associated with Alzheimer’s Disease. Inorg. Chem..

[16] Faller P. (2009). Copper and Zinc Binding to Amyloid-β: Coordination, Dynamics, Aggregation, Reactivity and Metal-Ion Transfer. ChemBioChem.

[17] Zou J., Kajita K., Sugimoto N. (2001). Cu^2+^ Inhibits the Aggregation of Amyloid β-Peptide(1-42) in Vitro. Angew Chem. Int. Ed. Engl..

[18] Yoshiike Y., Tanemura K., Murayama O., Akagi T., Murayama M., Sato S., Sun X., Tanaka N., Takashima A. (2001). New Insights on How Metals Disrupt Amyloid β-Aggregation and Their Effects on Amyloid-β Cytotoxicity *. J. Biol. Chem..

[19] Innocenti M., Salvietti E., Guidotti M., Casini A., Bellandi S., Foresti M.L., Gabbiani C., Pozzi A., Zatta P., Messori L. (2010). Trace Copper(II) or Zinc(II) Ions Drastically Modify the Aggregation Behavior of Amyloid-β 1-42: An AFM Study. J. Alzheimer’s Dis..

[20] Pedersen J.T., Østergaard J., Rozlosnik N., Gammelgaard B., Heegaard N.H.H. (2011). Cu(II) Mediates Kinetically Distinct, Non-Amyloidogenic Aggregation of Amyloid-β Peptides *. J. Biol. Chem..

[21] Atwood C.S., Moir R.D., Huang X., Scarpa R.C., Bacarra N.M.E., Romano D.M., Hartshorn M.A., Tanzi R.E., Bush A.I. (1998). Dramatic Aggregation of Alzheimer Aβ by Cu(II) Is Induced by Conditions Representing Physiological Acidosis *. J. Biol. Chem..

[22] Sarell C.J., Wilkinson S.R., Viles J.H. (2010). Substoichiometric Levels of Cu^2+^ Ions Accelerate the Kinetics of Fiber Formation and Promote Cell Toxicity of Amyloid-β from Alzheimer Disease *. J. Biol. Chem..

[23] Rajasekhar K., Madhu C., Govindaraju T. (2016). Natural Tripeptide-Based Inhibitor of Multifaceted Amyloid β Toxicity. ACS Chem. Neurosci..

[24] Reymond F., Fermín D., Lee H.J., Girault H.H. (2000). Electrochemistry at Liquid/Liquid Interfaces: Methodology and Potential Applications. Electrochim. Acta.

[25] Herzog G., Kam V., Arrigan D.W.M. (2008). Electrochemical Behaviour of Haemoglobin at the Liquid/Liquid Interface. Electrochim. Acta.

[26] Amemiya S., Yang X., Wazenegger T.L. (2003). Voltammetry of the Phase Transfer of Polypeptide Protamines across Polarized Liquid/Liquid Interfaces. J. Am. Chem. Soc..

[27] Trojánek A., Langmaier J., Samcová E., Samec Z. (2007). Counterion Binding to Protamine Polyion at a Polarised Liquid–Liquid Interface. J. Electroanal. Chem..

[28] Vagin M.Y., Malyh E.V., Larionova N.I., Karyakin A.A. (2003). Spontaneous and Facilitated Micelles Formation at Liquid|liquid Interface: Towards Amperometric Detection of Redox Inactive Proteins. Electrochem. Commun..

[29] Kivlehan F., Lanyon Y.H., Arrigan D.W.M. (2008). Electrochemical Study of Insulin at the Polarized Liquid−Liquid Interface. Langmuir.

[30] Scanlon M.D., Jennings E., Arrigan D.W.M. (2009). Electrochemical Behaviour of Hen-Egg-White Lysozyme at the Polarised Water/1, 2-Dichloroethane Interface. Phys. Chem. Chem. Phys..

[31] Abraham M.J., Murtola T., Schulz R., Páll S., Smith J.C., Hess B., Lindahl E. (2015). GROMACS: High Performance Molecular Simulations through Multi-Level Parallelism from Laptops to Supercomputers. SoftwareX.

[32] Oostenbrink C., Villa A., Mark A.E., Van Gunsteren W.F. (2004). A Biomolecular Force Field Based on the Free Enthalpy of Hydration and Solvation: The GROMOS Force-Field Parameter Sets 53A5 and 53A6. J. Comput. Chem..

[33] Hoover W.G. (1985). Canonical Dynamics: Equilibrium Phase-Space Distributions. Phys. Rev. A.

[34] Nosé S. (1984). A Molecular Dynamics Method for Simulations in the Canonical Ensemble. Mol. Phys..

[35] Nosé S., Klein M.L. (1983). Constant Pressure Molecular Dynamics for Molecular Systems. Mol. Phys..

[36] Parrinello M., Rahman A. (1981). Polymorphic Transitions in Single Crystals: A New Molecular Dynamics Method. J. Appl. Phys..

[37] Hess B. (2008). P-LINCS:  A Parallel Linear Constraint Solver for Molecular Simulation. J. Chem. Theory Comput..

[38] Darden T., York D., Pedersen L. (1993). Particle Mesh Ewald: An N⋅log(N) Method for Ewald Sums in Large Systems. J. Chem. Phys..

[39] Essmann U., Perera L., Berkowitz M.L., Darden T., Lee H., Pedersen L.G. (1995). A Smooth Particle Mesh Ewald Method. J. Chem. Phys..

[40] Crescenzi O., Tomaselli S., Guerrini R., Salvadori S., D’Ursi A.M., Temussi P.A., Picone D. (2002). Solution Structure of the Alzheimer Amyloid β-Peptide (1-42) in an Apolar Microenvironment. Eur. J. Biochem..

[41] Strodel B., Lee J.W.L., Whittleston C.S., Wales D.J. (2010). Transmembrane Structures for Alzheimer’s Aβ1−42 Oligomers. J. Am. Chem. Soc..

[42] Mutter S.T., Turner M., Deeth R.J., Platts J.A. (2020). Molecular Dynamics Simulations of Copper Binding to Amyloid-β Glu22 Mutants. Heliyon.

[43] Raffa D.F., Rauk A. (2007). Molecular Dynamics Study of the Beta Amyloid Peptide of Alzheimer’s Disease and Its Divalent Copper Complexes. J. Phys. Chem. B.

[44] Streltsov V.A., Titmuss S.J., Epa V.C., Barnham K.J., Masters C.L., Varghese J.N. (2008). The Structure of the Amyloid-β Peptide High-Affinity Copper II Binding Site in Alzheimer Disease. Biophys. J..

[45] Legleiter J., Thakkar R., Velásquez-Silva A., Miranda-Carvajal I., Whitaker S., Tomich J., Comer J. (2022). Design of Peptides That Fold and Self-Assemble on Graphite. J. Chem. Inf. Model..

[46] Touw W.G., Baakman C., Black J., te Beek T.A.H., Krieger E., Joosten R.P., Vriend G. (2015). A Series of PDB-Related Databanks for Everyday Needs. Nucleic Acids Res..

[47] Kabsch W., Sander C. (1983). Dictionary of Protein Secondary Structure: Pattern Recognition of Hydrogen-Bonded and Geometrical Features. Biopolymers.

[48] Balland V., Hureau C., Savéant J.-M. (2010). Electrochemical and Homogeneous Electron Transfers to the Alzheimer Amyloid-β Copper Complex Follow a Preorganization Mechanism. Proc. Natl. Acad. Sci. USA.

[49] Zhai J., Hoffmann S.V., Day L., Lee T.-H., Augustin M.A., Aguilar M.-I., Wooster T.J. (2012). Conformational Changes of α-Lactalbumin Adsorbed at Oil–Water Interfaces: Interplay between Protein Structure and Emulsion Stability. Langmuir.

[50] Jiang D., Rauda I., Han S., Chen S., Zhou F. (2012). Aggregation Pathways of the Amyloid β(1-42) Peptide Depend on Its Colloidal Stability and Ordered β-Sheet Stacking. Langmuir.

[51] Pedersen J.T., Heegaard N.H.H. (2013). Analysis of Protein Aggregation in Neurodegenerative Disease. Anal. Chem..

[52] Wofsy C., Goldstein B. (1992). Interpretation of Scatchard Plots for Aggregating Receptor Systems. Math. Biosci..

[53] Burgess A., Shah K., Hough O., Hynynen K. (2015). Focused Ultrasound-Mediated Drug Delivery through the Blood–Brain Barrier. Expert Rev. Neurother..

[54] Jun S., Gillespie J.R., Shin B., Saxena S. (2009). The Second Cu(II)-Binding Site in a Proton-Rich Environment Interferes with the Aggregation of Amyloid-β(1−40) into Amyloid Fibrils. Biochemistry.

