# Supplementary material for: An Electrochemistry and Computational Study at an Electrified Liquid–Liquid Interface for Studying Beta-Amyloid Aggregation
Source: Membranes (Basel). 2023 Jun 5;13(6):584. doi: 10.3390/membranes13060584 (PMC10303370; doi:10.3390/membranes13060584)
Supplement: Supplementary file 1 [file membranes-13-00584-s001.zip › membranes-2384505-SI.pdf]

Supplementary information

An Electrochemistry and Computational Study at an Electrified Liquid–Liquid Interface for Studying Beta-Amyloid Aggregation

[1]Department of Chemistry, Durham University, Durham, United Kingdom.

S1: Circular Dichroism Spectroscopy

An interfacial system was constructed in 1,2 DCE/water prepared as the same procedure in Scheme 1 using a glass vial. 5 µM of A$\beta_{(1\text{-}42)}$ was introduced in the upper aqueous phase, the system was incubated at room temperature without agitation. Three sets of experiments were carried out. Circular Dichroism spectra were collected for free A$\beta_{(1\text{-}42)}$, A$\beta_{(1\text{-}42)}$ in the presence of copper (II) ions and Cu- A$\beta_{(1\text{-}42)}$ in the presence of drug targeting A*β* aggregation and incubated for 24 hr. Spectra were obtained by wavelength scan from 260 to 190 nm using Jasco J-1500 CD spectrophotometer in 2 mm path length with a bandwidth of 1.0 nm, and three successive wavelength scans were averaged for each sample. The peptides were collected from the aqueous phase and measured at concentrations around 5 µM with all spectra being reported in ellipticity (mdeg).

Figure S-1 shows CD studies that were performed using samples removed from the aqueous phase of 1.2 DCE/ Aqueous interface containing the respective electrolytes, prepared to replicate the cell in scheme. Measurements were made after 24h incubation. CD spectroscopy was utilised in the range 260 to 190 nm to investigate the structural transition of the A$\beta_{(1\text{-}42)}$, Aβ_{42}-Cu(II) and Aβ_{42} -Cu(II) –P_6 samples. The CD spectrum of the free A$\beta_{(1\text{-}42)}$ exhibited peaks at approximately 197 nm characteristic for an unstructured conformation and a negative peak at approximately 2I7 nm characteristic of an Aβ sheet structure. These observations are consistent with previous reports and suggest that the Aβ plaques contain a mixture of amorphous and β-sheet aggregates.[1] In presence of Cu^{2+} ions there is an enhancement of both the negative and positive peaks, confirming an increase in aggregation. Upon incubation with P_6, the Aβ_{42}-Cu(II)-P_6 spectra displayed a drastic decrease in both the positive and negative peaks, which shows that the addition of P_6 inhibits the aggregation process.

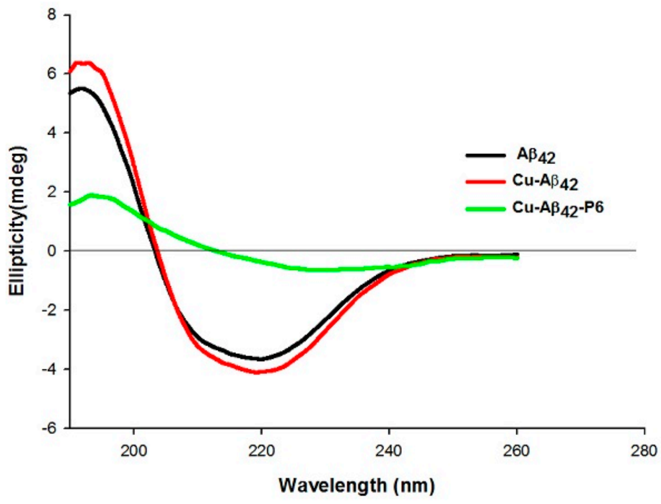

Figure S1: CD spectra of 5 µM (a) Aβ$_{(1-42)}$, (b) Aβ$_{(1-42}$ incubated 10 µM Cu(II) and (c) 15 µM of P_6 incubated with Cu(II) -Aβ$_{42}$ at a liquid liquid interfaces that was established with 1,2 Dichloroethane and lithium chloride for 24 hrs at room temperatures.

S2: Force field for dichloroethane (DCM)

GROMACS topology file for dichloroethane. Based on the GROMOS 53a6 paramter set.

```
[ moleculetype ]
; Name nrexcl
DCE 3
[ atoms ]
; nr type resnr resid atom cgnr charge mass total_charge
1 CH2 1 DCE C2 1 0.199 14.0270
2 CL 1 DCE CL1 1 -0.199 35.4530
3 CH2 1 DCE C1 1 0.199 14.0270
4 CL 1 DCE CL 1 -0.199 35.4530 ; 0.000
; total charge of the molecule: 0.000
[ bonds ]
; ai aj funct c0 c1
1 2 2 0.1820 1.6604e+06
1 3 2 0.1520 5.4300e+06
3 4 2 0.1820 1.6604e+06
[ pairs ]
; ai aj funct; all 1-4 pairs but the ones excluded in GROMOS itp
2 4 1
[ angles ]
; ai aj ak funct angle fc
2 1 3 2 114.00 1559.41
1 3 4 2 114.00 1559.41
[ dihedrals ]
; ai aj ak al funct ph0 cp mult
2 1 3 4 1 0.00 5.92 3
[ exclusions ]
; ai aj funct ; GROMOS 1-4 exclusions
```

S3: Structure of Aβ$_{(1\text{-}42)}$ in water: molecular dynamics simulation results.

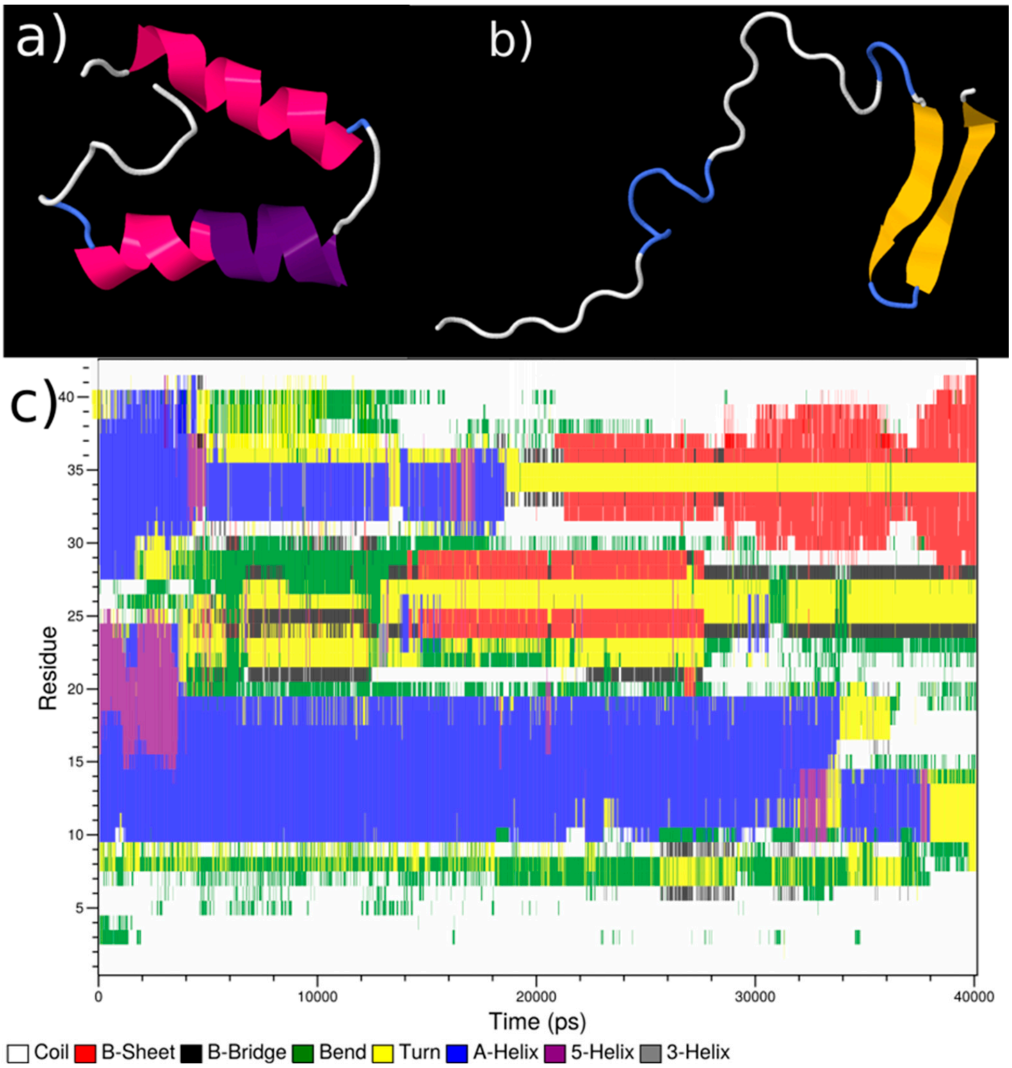

Figure S2 Equilibration of Aβ$_{(1\text{-}42)}$ in water, showing the formation of a β-hairpin structure. **a)** Snapshot of the pre-equilibrated PDB structure. **b)** Final structure from a 40 ns simulation in SPC water. **c)** Protein secondary structure during the equilibration of Aβ$_{(1\text{-}42)}$ in SPC water, determined using the DSSP software [6-7].

S4: Density functional theory structures for Cu(I) and Cu(II) binding sites.

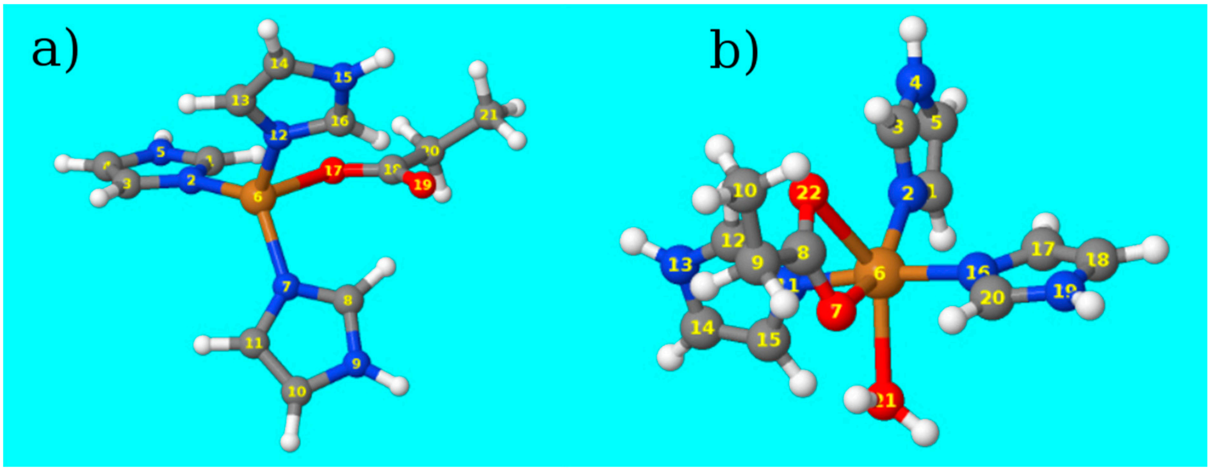

Figure S3: Optimised copper-binding sites for Cu(I) and Cu(II) sites, taken from B3LYP/LANL2DZ density functional theory calculations. The structures show the binding from the nitrogen on His residues (His13, His14, His6) and a carboxylic acid group (from an Asp or Glu residue). **a)** Cu(I) distorted octahedral binding site for neutral Cu^{1+} complex (singlet). **b)** Cu(II) binding site for +1 charged complex of Cu^{2+} (doublet) with one coordinate site taken by a water molecule. The structure in (b) is identical to the calculation by Streitsov et al. [8], which is supported by EXAFS results. On addition of 1e- to the Cu(II) binding site in (b), energy minimisation immediately transforms the binding site to structure (a) via ejection of the water molecule, which hydrogens to oxygen 17.

S5: Molecular dynamics structure of the copper (II) binding site and additional force field parameters for copper binding

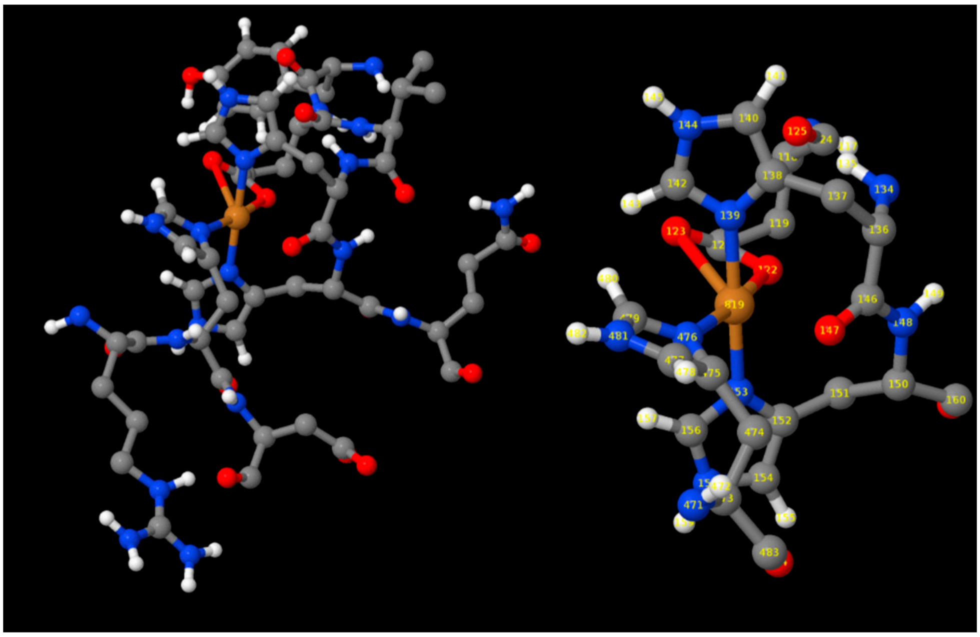

Figure S4: Molecular dynamics distorted octahedral binding site for Cu(II). In this snapshot, the carbonyl carbon (atom 147) from residue HIS13 (chain 1) is conveniently placed to occupy the vacant 6th coordination site (contact distance 0.233 nm), which can be taken by a water molecule. Here the other copper coordinated atoms are atom 139 (HIS13, chain 1), atom 153 (HIS14, chain 1), atom 476 (HIS6, chain 2), atoms 122, 123 (GLU11, chain 1).

Table S1: Additional bond lengths (LINCS bond constraints used)

Atom	Atom	Bond length / nm
N(His)	Cu^{2+}	0.203
O(Glu) short bond	Cu^{2+}	0.206
O(Glu) long bond	Cu^{2+}	0.259

Table S2: Additional bond angle parameters

Atom	Atom	Atom	θ^0/ °	k^θ / kJ mol^{-1} rad^{-2}
N(His13)	Cu^{2+}	N(His14)	180.0	562.16
N(His, other)	Cu^{2+}	N(His, other)	90.0	562.16
N(His6)	Cu^{2+}	O(OE1, Glu11)	180.0	562.16
N(His, other)	Cu^{2+}	O(OE1, Glu11)	90.0	562.16
C(His)	N(His)	Cu^{2+}	126.2	562.16
O(OE1, Glu11)	C(Glu)	Cu^{2+}	102.7	562.16
O(OE2, Glu11)	C(Glu)	Cu^{2+}	80.4	562.16

Table S3: Partial charges

Atom	Non-copper containing peptide Partial charge / e^-	Copper containing peptide Partial charge / e^-
[a]Cu^{2+}	---	1.2
N(His)	-0.54	-0.38
O(Glu11)	-0.635	-0.475

[a]Following previous work [9], the +2 charge on Cu^{2+} is reduced to 1.2 (partially delocalized to the coordinated atoms). The partial charge of 1.2 on the copper is in excellent agreement with partial charge on the copper (1.1965) obtained by fitting to the electrostatic potential using the CHELPG scheme and density functional theory calculations at the B3LYP/LANL2DZ level.

S6: Summary of systems simulated with molecular dynamics

Table S4: Summary of systems simulated with molecular dynamics

System	Peptide(s)	copper	water	Na+	Cl-	DCE	Time / ns	notes
A	1	---	36681	105	102	1960	50.0	Peptide "captured" by interface
B	4	---	37028	216	204	7840	303.3	Interface 6.25 nm x 6.16 nm
C	4	---	20400	216	204	4319	320.0	Interface 9.51 nm x 9.52 nm
D	2	---	20400	208	202	4319	1365.2	Interface 9.95 nm x 9.94 nm
E	2	Cu^{1+}	20400	208	203	4319	1665.8	Interface 9.95 nm x 9.94 nm
F	2	Cu^{2+}	20400	207	203	4319	1239.7	Interface 9.95 nm x 9.94 nm

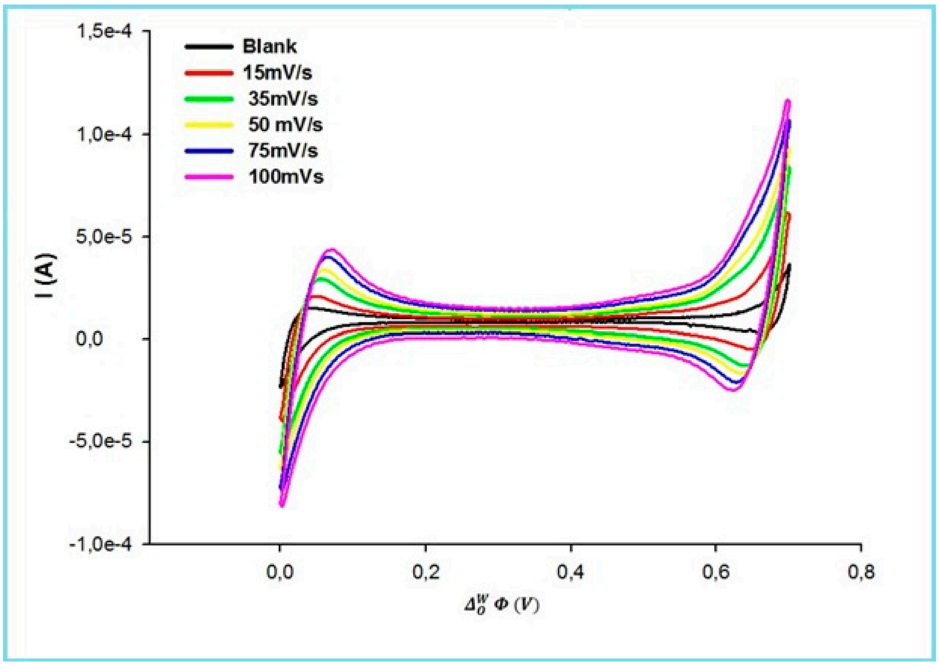

Figure: S5: Cyclic voltammograms of scan rate studies of Aβ$_{(1-42)}$ at ITIES, using Cell2 Scheme 2

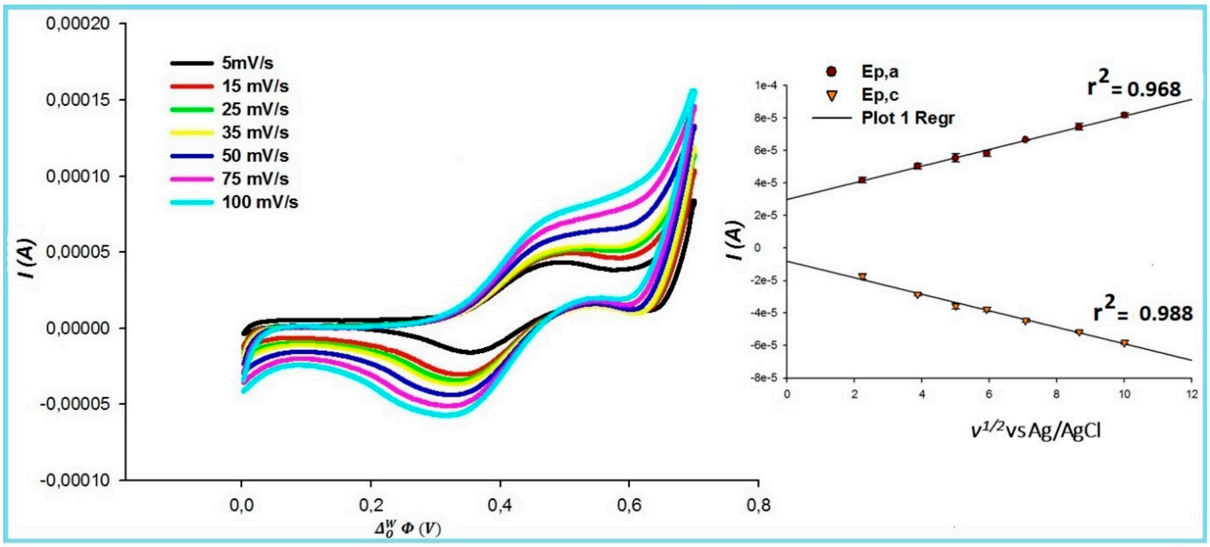

Figure: S6: Cyclic voltammograms of scan rate studies of Cu- Aβ$_4$ at ITIES, inset plot of positive and negative current vs square root of various scan rate, using a linear least square fitting of the peak c urrents

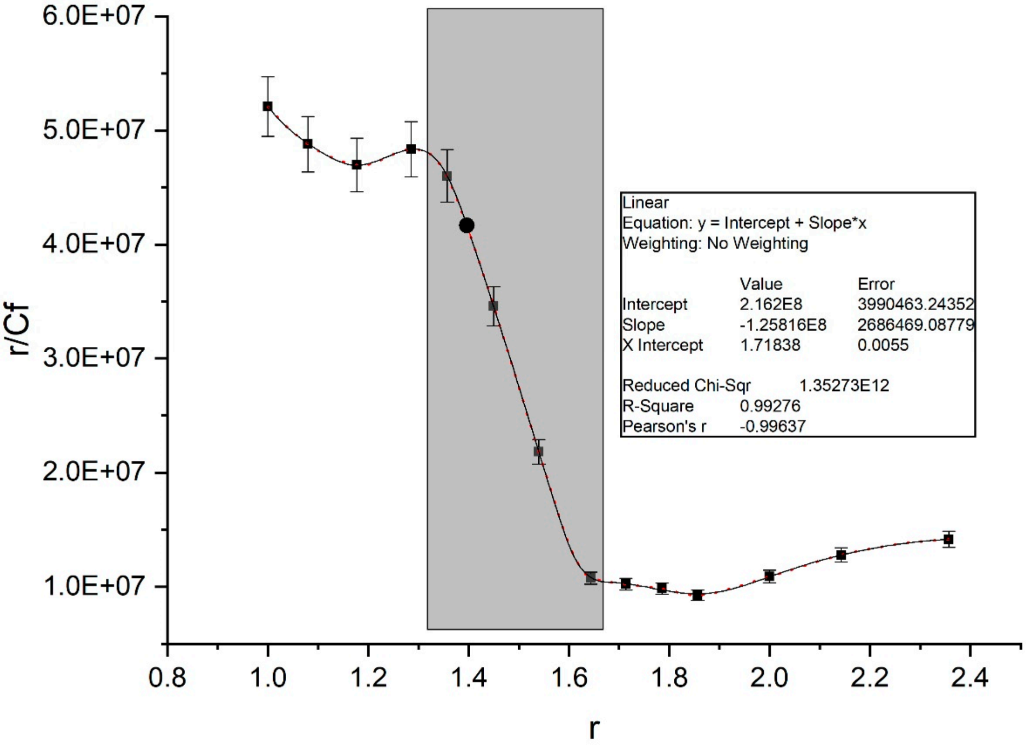

Figure: S7 Scatchard plot showing the interactions of Cu(II) with Aβ(1-42). at ITIES. The most significant binding constant was obtained from the linear portion of the graph shown in the box.

Apparent association constant of Aβ(1-42) with Cu (II) are evaluated using Scatchard equation: [4,5]

$$\frac{r}{C_F} = nK - \boldsymbol{r}\,K \;(1)$$

$$r = \frac{C_b}{[A\beta_{1-42}]} \;(2)$$

Where, K is the apparent association constant, r is a binding ratio, C_F is a free concentration of Cu(II) ions and n is the binding site. Inset shows the DPVs on addition of Cu(II) concentration to Aβ(1-42).

S7: Protein dimer aggregation at a dichloroethane-water interface: snapshots structures

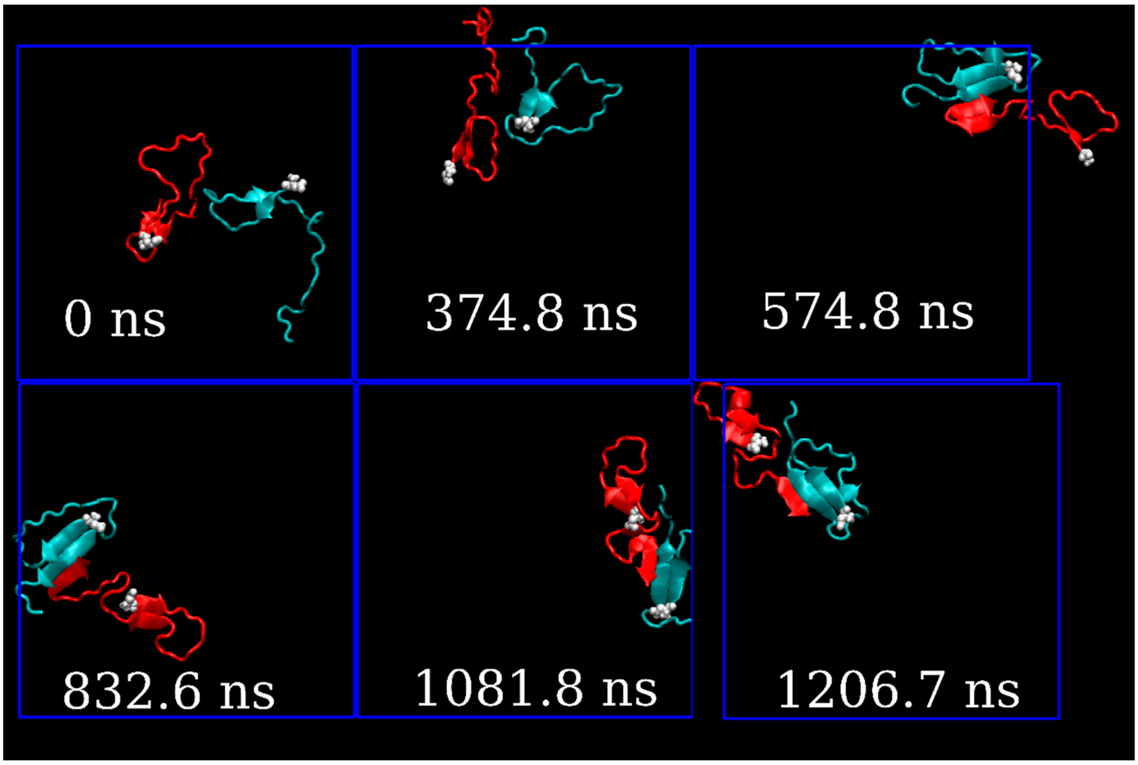

Figure S8a: Association of the protein dimer at the dicholoroethane-water. The time-sequence shows the dimer without copper. Individual chains are colour-code in cyan and red, and the first residue of each chain is shown in white. (Simulation box cross-section 9.95 nm x 9.94 nm.)

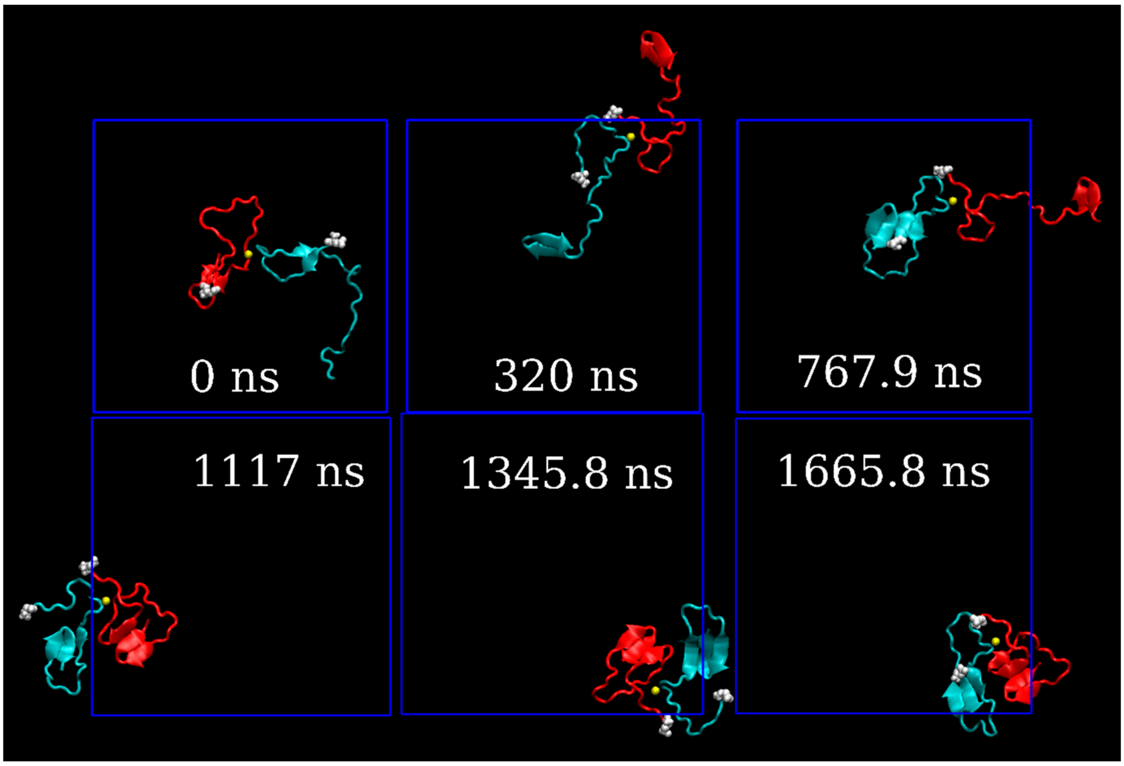

Figure S8b: Association of the protein dimer at the dicholoroethane-water. The time-sequence shows the dimer with a copper (I) ion bound (as shown in Figure S-3a). Individual chains are colour-code in cyan and red, the copper ion is shown in yellow and the first residue of each chain is shown in white. (Simulation box cross-section 9.95 nm x 9.94 nm.)

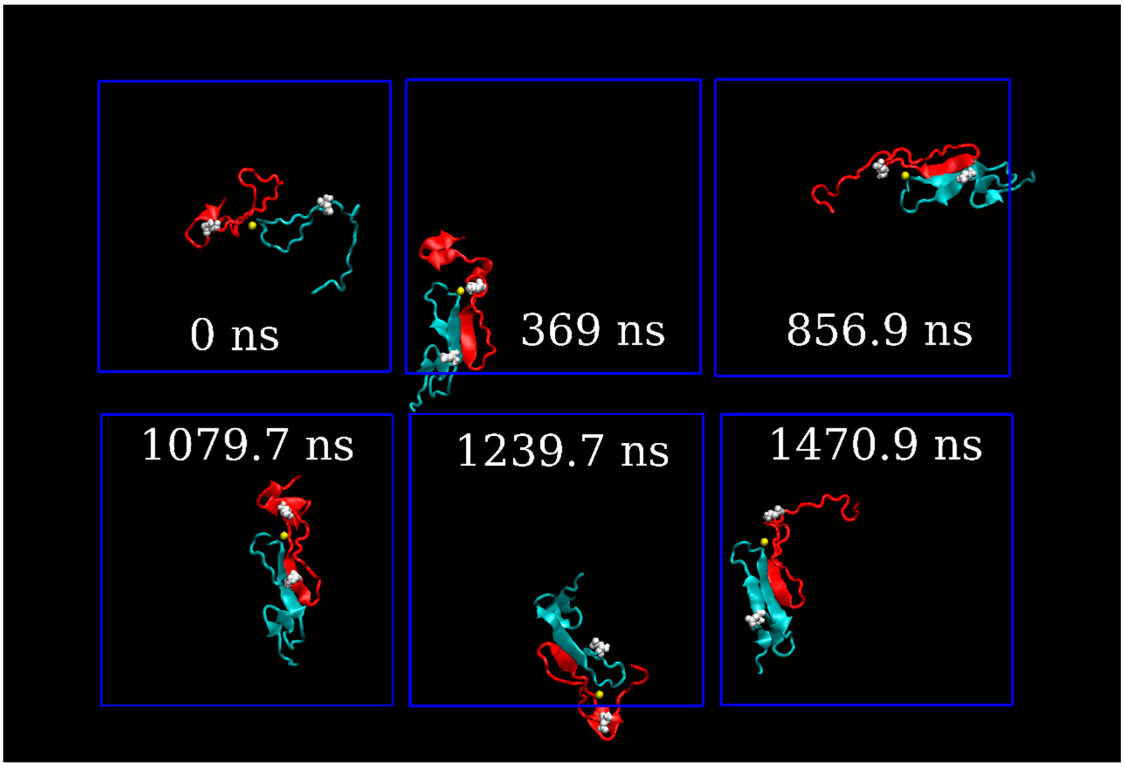

Figure S8c: Association of the protein dimer at the dicholoroethane-water. The time-sequence shows the dimer with a copper (II) ion bound (as shown in Figure S-3b). Individual chains are colour-code in cyan and red, the copper ion is shown in yellow and the first residue of each chain is shown in white. (Simulation box cross-section 9.95 nm x 9.94 nm.)

S8: Protein tetramer aggregation at a dichloroethane-water interface: snapshots at low surface concentration.

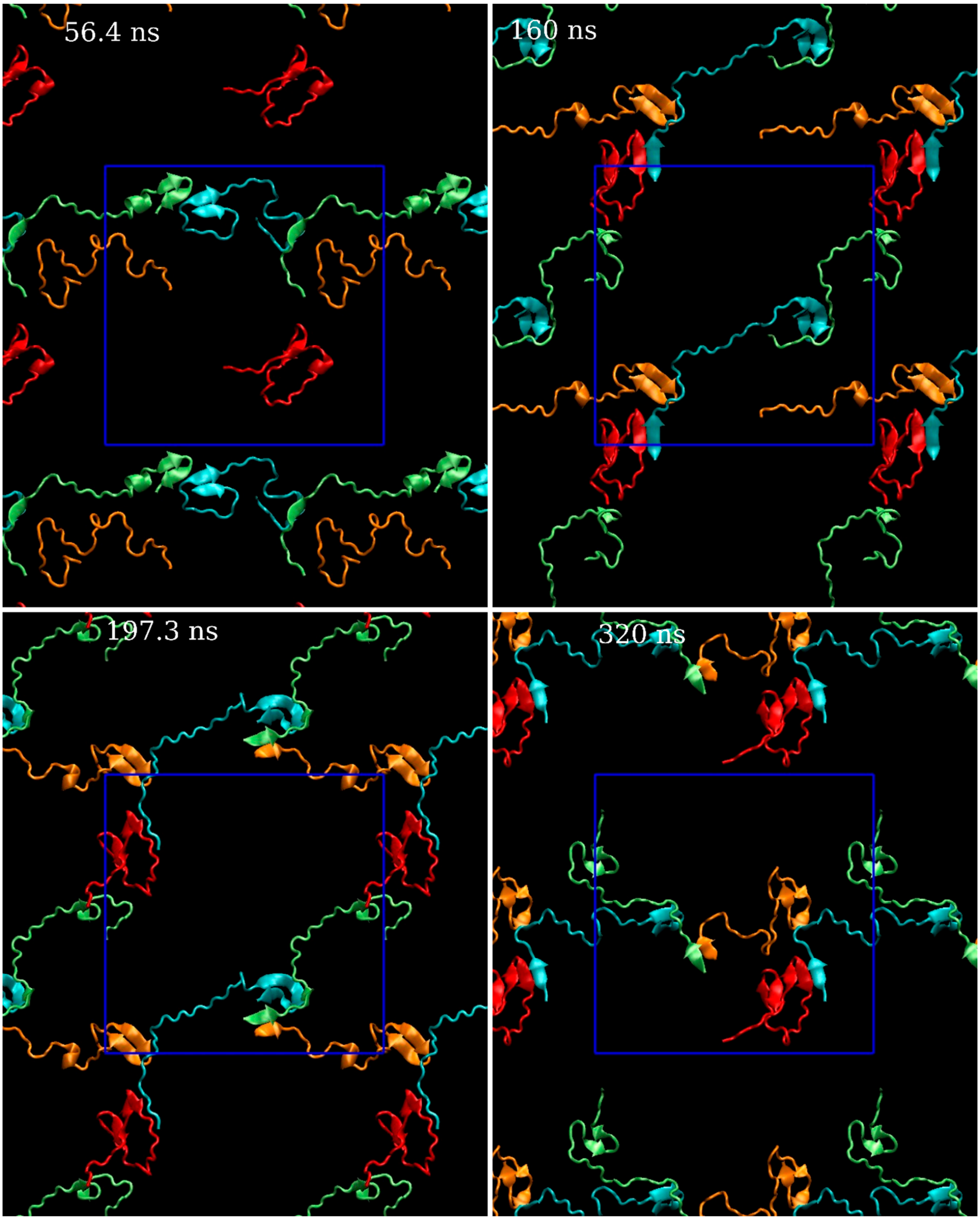

Figure S9: Snapshots showing aggregation of A$\beta_{1\text{-}42}$ peptides at a DCE-water interface. Four chains are shown in cyan, red, lime green and orange together with protein secondary structure. The dark blue box outline indicates the 2d-profile of the periodic box, which shows an average dimension of ~9.51 nm x 9.52 nm.

References

(1) Dr Neil Rees , Dr Sarah Horswell , Dr Paramaconi Rodriguez , Professor Alison Davenport University of Birmingham. 1–143.

(2) Cheignon, C.; Jones, M.; Atrián-Blasco, E.; Kieffer, I.; Faller, P.; Collin, F.; Hureau, C. Identification of Key Structural Features of the Elusive Cu–Aβ Complex That Generates ROS in Alzheimer's Disease. *Chem. Sci.* **2017**, *8* (7), 5107–5118.

(3) Ma, Q.F.; Hu, J.; Wu, W.H.; Liu, H.; Du, J.T.; Fu, Y.; Wu, Y.W.; Lei, P.; Zhao, Y.F.; Li, Y.M. *Biopolymers* **2006**, 83, 20–31

(4) Scatchard, G. The Attraction of Proteins for Small Molecules and Ions. *Annals of the New York Academy of Sciences* **2006,** 51(4):660–672.

(5) Viljan, L. E. Absorption studies of oflaxacin-DNA system. *Optoelectronics and advanced materials-rapid communication.* **2010**, *3* (1), 60–64.

(6) Wouter G Touw, Coos Baakman, Jon Black, Tim AH te Beek, E Krieger, Robbie P Joosten, Gert Vriend. A series of PDB related databases for everyday needs. *Nucleic Acids Research* **2015**, 43: D364-D368.

(7) Kabsch W, Sander C. Dictionary of protein secondary structure: pattern recognition of hydrogen-bonded and geometrical features. *Biopolymers* **1983**, 22: 2577-2637.

(8) Streltsov Victor A., Titmuss Stephen J., Epa V. Chandana, Barnham Kevin J., Masters Colin L., and Varghese Joseph N. The Structure of the Amyloid-*β* Peptide High-Affinity Copper II Binding Site in Alzheimer Disease. *Biophys J.* **2008**, 95(7): 3447–3456.

(9) Raffa D. F. and Rauk A. Molecular Dynamics Study of the Beta Amyloid Peptide of Alzheimer's Disease and Its Divalent Copper Complexes. *J. Phys. Chem B*, **2007** *111* (14), 3789-3799.
